# Transcriptional upregulation of CXCL13 is correlated with a favorable response to immune checkpoint inhibitors in lung adenocarcinoma

**DOI:** 10.1002/cam4.5460

**Published:** 2022-12-01

**Authors:** Sehhoon Park, Hongui Cha, Hong Sook Kim, Boram Lee, Soyeon Kim, Tae Min Kim, Hyu Ae Jung, Jong‐Mu Sun, Jin Seok Ahn, Myung‐Ju Ahn, Keunchil Park, Woong‐Yang Park, Se‐Hoon Lee

**Affiliations:** ^1^ Division of Hematology‐Oncology, Department of Medicine, Samsung Medical Center Sungkyunkwan University School of Medicine Seoul Republic of Korea; ^2^ Department of Health Science and Technology, Samsung Advanced Institute of Health Science and Technology Sungkyunkwan University Seoul Republic of Korea; ^3^ Samsung Genome Institute Samsung Medical Center, Sungkyunkwan University School of Medicine Seoul Republic of Korea; ^4^ Seoul National University Cancer Research Institute Seoul Republic of Korea; ^5^ Biomedical Research Institute, Seoul National University Hospital Seoul Republic of Korea; ^6^ Departments of Internal Medicine Seoul National University Hospital, Seoul National University College of Medicine Seoul Republic of Korea

**Keywords:** adenocarcinoma, CXCL13, immune‐checkpoint inhibitor, non‐small cell lung cancer, tertiary lymphoid structure

## Abstract

**Background:**

The chemokine *CXCL13* is known to influence local anti‐tumor immunity by recruiting immune cells and forming tertiary lymphoid structures (TLS). It has been hypothesized that TLS, led by the expression of *CXCL13*, could be a predictive or prognostic biomarker for immunotherapy. We investigated the predictive value of *CXCL13* to immune checkpoint inhibitors (ICI) in lung adenocarcinoma.

**Methods:**

We constructed an exploratory dataset (*n* = 63) and a validation dataset (*n* = 57) in metastatic lung adenocarcinoma patients treated with ICI. Based on the clinical response, the difference in gene expression profile, including CXCL13, was evaluated.

**Results:**

From the exploratory dataset, *CXCL13* expression was significantly upregulated in the ICI responders (*p* = 0.002). Survival analysis using a cut‐off value of the median expression value of *CXCL13* showed prolonged progression‐free survival (PFS) (*p* = 0.004) and overall survival (OS) (*p* = 0.007). *CXCL13* expression was correlated with other immune response genes, such as *GZMA*, *CD8A*, *IFNG*, *PRF1*, TLS‐related gene sets and its receptor, *CXCR5*. Notably, subgroup analyses based on *CXCL13* expression and *CD8A* showed that *CXCL13‐*upregulated patients demonstrated comparably prolonged survival regardless of *CD8A* expression. In the validation dataset, *CXCL13* upregulation also demonstrated a significant prolongation of both PFS (*p* = 0.050) and OS (*p* = 0.026).

**Conclusion:**

We observed that *CXCL13* upregulation is correlated to better ICI response in lung adenocarcinoma. Our results support that CXCL13 could be an important chemokine in shaping the immunoactive tumor microenvironment which affects the anti‐tumor effect of ICI.

## Graphical abstract

*CXCL13* expression in lung adenocarcinoma showed predictive value to immune checkpoint inhibitor (ICI) treatment. *CXCL13* expression was correlated with expression of other immune response genes. Higher median progression‐free survival and overall survival to ICI treatment are observed in *CXCL13* upregulated group.

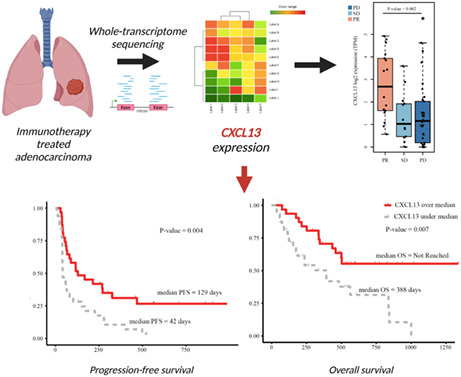

## 1 BACKGROUND

The programmed death‐ligand 1 (PD‐L1) and PD‐1 inhibitor have shown prolonged response as either monotherapy or in combination with cytotoxic chemotherapy in non‐small cell lung cancer (NSCLC), and adenocarcinoma.^1^, ^2^ Current research on the predictive biomarker of immune checkpoint inhibitors (ICIs) is focused on tumor characteristics namely the neoantigen burden calculated from somatic mutation,^3^, ^4^, ^5^ expression of PD‐L1,^6^ tumor microenvironment evaluated by infiltration of cytotoxic T cells,^7^, ^8^ and functional signatures related to cytotoxic activity.^9^

The presence of the tertiary lymphoid structure (TLS), ectopic lymphoid tissue composed of multiple immune cells that mimic the structure and the function of the secondary lymph node, has been discussed as a potential exploratory biomarker for the immunotherapy.^10^, ^11^ TLS is known to hinder tumor adaptive immunity by functioning as a privileged site of tumor antigen presentation to the T‐cell mostly by dendritic cells.^12^ Based on this observation, it is hypothesized that the role of TLS could be as an immune inductive site against cancer which generates tumor‐specific effector cells and induces the migration to the tumor cells.^13^ In support of this hypothesis, research on the presence of TLS in NSCLC showed not only a strong correlation with the infiltration of cytotoxic T‐cells in quantity but also that TLS affected the function of tumor‐infiltrating lymphocytes (TILs). It is demonstrated in the patients with a high density of TILs without TLS showed poor prognosis compared to patients with a high density of TILs with TLS who showed a favorable clinical outcome.^12^

It is reported that the formation of TLS is initiated by CXC‐chemokine ligand 13 (CXCL13), which is locally produced by various lymphocyte and stromal cell, by recruiting the lymphoid tissue inducer cells and promote high endothelial venules.^14^, ^15^ It is notable that CXCL13 is also the key component of various TLS gene signatures reported in different types of cancers.^16^, ^17^ As related to the clinical efficacy of immunotherapy, the expression of *CXCL13* was also identified in a unique subset of PD‐1 highly cytotoxic T cells in NSCLC, which showed an association with ICI response^18^ Moreover, the correlation between *CXCL13* expression and ICI response is also reproduced in other types of cancer.^19^, ^20^, ^21^, ^22^

As the *CXCL13* is observed as the constituent member of the well‐described chemokine gene signature associated with TLS formation^17^, ^23^, ^24^, ^25^ and also well associated with the favorable outcome in pan‐cancer analysis,^26^ we hypothesized that the investigating the transcriptional upregulation of *CXCL13*, consider it as a surrogated marker of TLS, could have a correlation with favorable predictive value in the response of lung adenocarcinoma to ICIs and known genes related to the ICI response.

## 2 METHODS

### 2.1 Patient selection: clinical and histological information

Samples from patients treated with either PD‐1 or PD‐L1 inhibitors between August 2014 and February 2019 at Samsung Medical Center or Seoul National University Hospital were retrospectively and prospectively collected. For the patient selection, we mandated the below criteria for the study participation (a) patients confirmed with metastatic non‐small cell lung cancer with adenocarcinoma histology; (b) received at least one cycle of immune checkpoint inhibitor treatment; (c) available for the response evaluation; (d) who agree to provide the sample for the study or consent waived in accordance to the ethical board guideline; (e) tissue sufficient for the whole transcriptomic sequencing (WTS) analysis. For the exclusion criteria, those who had previously received an immune checkpoint inhibitor before the sample collection were excluded. To evaluate the tissue availability, each specimen was evaluated by the pathologist for the assessment of purity and the size of the tumor. At least 15 and 5 slides for tumors with sizes below 1 × 1 cm and over 1 × 1 cm, respectively, were prepared for the RNA isolation. 4 um unstained slide was constructed from the formalin‐fixed paraffin‐embedded (FFPE) onto positively charged slides. For the exclusion criteria, those who has previously received immunotherapy before the sample collection were excluded. Based on the pre‐defined criteria, all the patients included were histologically confirmed for adenocarcinoma.

The WTS data were generated from 63 patients using an Access kit and used as an exploratory dataset. The data from 57 patients with WTS results determined by using a TruSeq kit were used as a validation dataset. Clinical information, including baseline characteristics, types of treatment, and treatment outcomes, was collected from patients' electronic medical records. In addition, PD‐L1 immunohistochemistry (IHC) results were recorded based on tumor proportional score (TPS) using the 22C3 pharmDx antibody (Agilent, USA) and were also extracted from the electronic medical records.

This study was conducted with the approval of an institutional review board (IRB number: 2018–03‐130 and 2013–10‐112), and informed consent was obtained from the patients who agreed to provide the sample for the analysis. For those who were not available for the signed consent, informed consent was waived in accordance with the IRB guidelines.

### 2.2 Genomic RNA preparation and whole transcriptome sequencing

RNA was purified from formalin‐fixed paraffin‐embedded (FFPE) or fresh tumor samples using the AllPrep DNA/RNA Mini Kit (Qiagen, USA). RNA concentration and purity were measured using the NanoDrop and Bioanalyzer (Agilent, USA). The library was prepared following the manufacturer's instructions using either the TruSeq RNA Library Prep Kit v2 (Illumina, USA) or the TruSeq RNA Access Library Prep Kit (Illumina, USA). Isolated total RNA was used in a reverse transcription reaction with poly (dT) primers, using SuperScript TM II Reverse Transcriptase (Invitrogen/Life Technologies, USA), according to the manufacturer's protocol. An RNA‐seq library was prepared via cDNA amplification, end‐repair, 3′ end adenylation, adapter ligation, and amplification. Library quality and quantity were measured using the Bioanalyzer and Qubit. Sequencing was performed on the HiSeq 2500 platform (Illumina, USA). The read from FASTQ files was mapped against the human genome (hg) 19 using the 2‐pass mode of STAR version 2.4.0. Raw read counts mapped to genes were analyzed for transcript abundance using RSEM version 1.2.18, and poorly expressed samples were eliminated based on the criteria of the read count <1 M. For the normalization between the sample, we used transcript per million (TPM) values that consider sequencing depth and gene length.

### 2.3 Differentially expressed gene analysis, tumor purity, and tumor mutation burden (TMB) calculation

A differentially expressed gene (DEG) analysis, using 271 immune‐related genes,^27^, ^28^ was conducted between patients showing partial response and either stable disease or progressive disease evaluated by response evaluation criteria in solid tumor v1.1 using the Mann–Whitney test with gene sets represent the immune landscape of cancer. Based on pre‐defined criteria, a 1.5‐fold difference in expression between the groups analyzed by a nominal two‐sided Mann–Whitney (*p*‐value <0.05) was used as a cut‐off value for significance. Tumor purity was calculated using ESTIMATE. To calculate the TMB, a library was created following the manufacturer's instructions for SureSelectXT Human All Exon V5, and sequencing was performed on the HiSeq 2500 platform (Illumina, USA). Target coverage was 50x for the normal control samples and 100x for the tumor samples. The sequencing data were aligned to the hg19 human genome. Mutations were annotated using MuTect for somatic mutations. TMB was measured by the total number of mutations except for synonymous mutations, per Mb.

### 2.4 Gene sets of interest

We used multiple gene sets reported to be related to the cytolytic activity, Immunoscore (http://www.immunoscore.org/research.shtml), cytolytic (CYT) score,^29^ gene expression profile (GEP),^30^ and gene expression markers of tumor‐infiltrating lymphocytes.^27^ The gene set of TLSs was used from previous reports.^31^, ^32^

### 2.5 The cancer genome atlas (TCGA) data

We obtained WTS data of normal lung and lung adenocarcinoma from the Broad GDAC firehose level 3 (https://gdac.broadinstitute.org/). Clinical data were acquired from the cbioportal (http://www.cbioportal.org/datasets). In total, 515 tumors and 59 normal samples were available for the analyses. The expression profile of *CXCL13* and immune‐related genes were compared using the Pearson correlation method. Survival analysis was conducted using available clinical information.

### 2.6 Statistical analysis

Clinical outcome is categorized based on the Response Evaluation Criteria for Solid Cancer. Kaplan–Meier survival curves were used to estimate the patterns of progression‐free survival (PFS) and overall survival (OS). The PFS was defined as the date from the ICI treatment to the date of confirmed disease progression or death event. Similarly, the OS was defined as the date from the ICI treatment to the date of death event. The log‐rank test was used to calculate *p*‐values. Correlations were determined using the Pearson correlation method; the Mann–Whitney test was used to compare the difference between the two groups, and the Kruskal‐Wallis test was used to compare the differences between the three groups. All statistical analyses were done in the R‐3.6.0 program for Windows. *P‐*values less than 0.05 were considered significant.

## 3 RESULTS

### 3.1 Patient characteristics

Basic demographic profiles were similar between the exploratory and validation datasets (Table 1). Patients in the exploratory and validation datasets were treated with pembrolizumab (69.4% and 49.1%), nivolumab (36.5% and 31.6%), and atezolizumab (12.7% and 7.0%), respectively. Treatments were mostly applied as third‐line or beyond: the exploratory dataset (58.7%) and the validation dataset (66.7%). Samples used for the WTS were mainly acquired from the lung parenchymal tissue (33.3%) and lymph nodes (69.4%) for the exploratory dataset and lung (35.1%) and LN (21.1%) for the validation dataset. High PD‐L1 expression patients defined by TPS PD‐L1 IHC greater or equal to 50% were 36.5% in the exploratory dataset and 42.1% in the validation dataset.

**TABLE 1:** Baseline characteristics of the study population

		Exploratory dataset ‐Access kit (*n* = 63)	Validation dataset ‐TruSeq kit (*n* = 57)
Age	Median (Range)	59 (40–85)	57 (33–81)
Sex			
	Male	40 (63.5%)	38 (66.7%)
	Female	23 (36.5%)	19 (33.3%)
Smoking history			
	Never smoker	29 (46.0%)	21 (36.8%)
	Ex‐smoker	11 (17.5%)	18 (31.6%)
	Current smoker	23 (36.5%)	18 (31.6%)
Histology			
	Adenocarcinoma	64 (100.0%)	57 (100.0%)
Molecular subtypes			
	Activating EGFR mutation	7 (11.1%)	19 (33.3%)
	ALK rearrangement	3 (4.8%)	0 (0.0%)
ECOG PS			
	0	0 (0.0%)	2 (3.5%)
	1	56 (88.9%)	48 (84.2%)
	2	7 (11.1%)	7 (12.3%)
Previous RT history			
	*n*	24 (38.1%)	20 (35.1%)
PD‐L1 staining (DAKO 22C3)			
	TPS ≥ 50%	23 (36.5%)	24 (42.1%)
	1% ≤ TPS < 50%	12 (19.0%)	12 (21.1%)
	TPS <1%	16 (25.5%)	11 (19.3%)
	Not available	12 (19.0%)	10 (15.8%)
Data available for tumor mutation burden			
	*n*	41 (65.1%)	52 (91.2%)
Backbone ICI			
	Pembrolizumab	25 (69.4%)	28 (49.1%)
	Nivolumab	23 (36.5%)	18 (31.6%)
	Atezolizumab	8 (12.7%)	4 (7.0%)
	Durvalumab	2 (3.2%)	3 (5.3%)
	Avelumab	5 (7.9%)	3 (5.3%)
	Other	0 (0.0%)	1 (1.8%)
ICI treatment line			
	1 L	4 (6.3%)	4 (7.0%)
	2 L	23 (36.5%)	15 (26.3%)
	≥3 L	36 (57.1%)	38 (66.7%)
Biopsy sites			
	Lung	21 (33.3%)	20 (35.1%)
	Lymph node	25 (69.4%)	12 (21.1%)
	Other	17 (27.0%)	25 (43.9%)

Abbreviations: ECOG PS, Eastern cooperative oncology groups performance score; ICI, immune checkpoint inhibitor; TPS, tumor proportion score.

### 3.2 *CXCL13* is significantly upregulated in the ICI responder in the exploratory dataset

**FIGURE 1:**
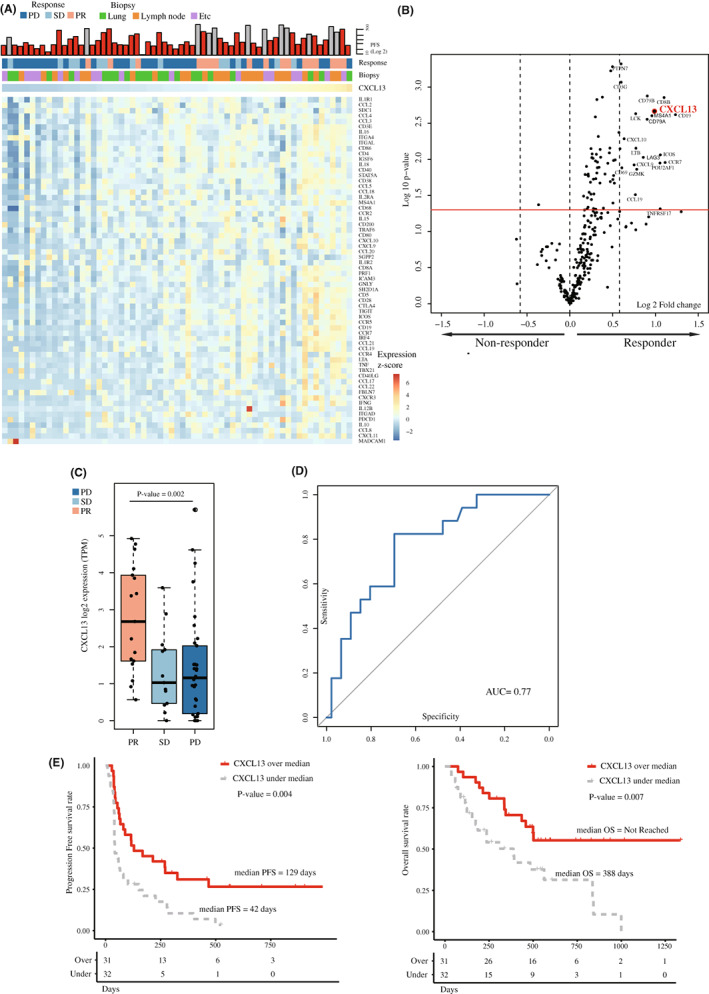
(A) Heatmap showing *CXCL13* and gene set related to the tertiary lymphoid structure. (B) Volcano plot of differentially expressed genes showing significant changes between immune checkpoint inhibitor (ICI) responder versus non‐responders. Analyses were conducted with the exploratory dataset (*n* = 63) (C) TPM of *CXCL13* based on the best overall response to ICI. (D) Predictive value of *CXCL13* to ICI. (E) Progression‐free survival and the overall survival using the cutoff value of median *CXCL13* expression profile.

From our exploratory dataset, *CXCL13* showed a similar expression pattern to the previously reported gene set of TLS (Figure 1A). In the DEG conducted with a pre‐defined gene set related to the immune landscape, *CXCL13* showed 1.97‐fold changes (*p* = 0.002) in the responder (Figure 1B, Table 2). The DEG profile identified 21 genes significantly upregulated in patients with ICI responders compared to those with non‐responders (Figure S1A). To evaluate the potential bias based on biopsy sites, we conducted analyses between the lung, lymph node and other tissue. Tumor purity calculated by ESTIMATE showed no difference between the lung and lymph nodes (*p* = 0.991) (Figure S1B). No difference in *CXCL13* expression was also observed between biopsy sites, lung, and lymph nodes (*p* = 0.251). The median TPM of *CXCL13* in partial response patients was 5.41 (95% confidential interval [CI] 0.48–29.38) which is significantly higher than the TPM of patients with stable disease (SD), median 1.04 (95% CI 0.00–11.07) and progressive disease (PD), median 1.28 (95% CI 0.00–51.10) (Figure 1C). Using the cut‐off value as a median TPM of *CXCL13*, there was no significant difference in baseline characteristics between the *CXCL13* high and low groups. Using the median TPM as the cut‐off value, the area under curve (AUC) was 0.77 (Figure 1D). This is similar to the AUC conducted with other gene sets such as Immunoscore (AUC = 0.75), CYT score (AUC = 0.65), GEP (AUC = 0.75), CTL (AUC = 0.70), and Danaher et al. (AUC = 0.74) (Figure S1C). There was no incrementation in predictive value when *CXCL13* was combined with other gene sets (Figure S2A). Survival analysis showed significantly longer PFS (*p* = 0.004) and OS (*p* = 0.007) in patients with high *CXCL13* expression using cut‐off value as median TPM (Figure 1 E, Table 3).

**TABLE 2:** Clinical outcomes of the study population

		Exploratory dataset ‐Access kit (*n* = 63)	Validation dataset ‐TruSeq kit (*n* = 57)
Overall response rate		27.0%	29.8%
Overall response			
(*n*)	PR	17 (27.0%)	17 (29.8%)
	SD	13 (20.6%)	10 (17.5%)
	PD	33 (52.4%)	30 (52.6%)
Tumor purity		*p* = 0.991^a^	*p* = 0.991^a^
Median (range)	Lung	0.67 (0.34–0.79)	0.70 (0.21–0.80)
	Lymph node	0.63 (0.34–0.84)	0.63 (0.52–0.77)
	Other	0.69 (0.37–0.93)	0.67 (0.34–0.95)
TPM of CXCL13 based on clinical response		*p* = 0.002*	*p* = 0.024*
Median (range)	PR	5.41 (0.48–29.38)	1.72 (0.00–33.10)
	SD	1.04 (0.00–11.07)	1.64 (0.00–32.98)
	PD	1.29 (0.00–51.10)	0.98 (0.00–35.64)
TPM of CXCL13 based on biopsy sites		*p* = 0.251^a^	*p* = 0.107^a^
Median (range)	Lung	1.16 (0–5.70)	1.24 (0–5.20)
	Lymph node	1.88 (0–4.78)	3.00 (0–5.09)
	Other	0.95 (0–4.93)	0.78 (0–4.98)
Median tumor mutation burden		*p* = 0.784*	*p* = 0.051*
	PR	2.34 (0.48–66.28)	2.34 (0.16–27.15)
	SD	2.40 (0.54–8.61)	2.32 (0.42–3.89)
	PD	2.34 (0.34–12.24)	2.26 (0.50–7.52)
Progression‐free survival (days)	Median (95% CI)	67 (54–168)	73 (47–119)
Overall survival (days)	Median (95% CI)	500 (344 ‐ NR)	338 (240–606)

*Note*: Tumor purity is calculated by ESTIMATE.

Abbreviations: NR, not reached; PD, progressive disease; PR, partial response; SD, stable disease; TPM, transcripts per kilobase million.

* *p*‐value calculated by two‐sided Mann–Whitney test by comparing PR patients to the SD or PD patients.

^a^ *p* value calculated by two‐sided Mann–Whitney test by comparing the value from lung and lymph nodes.

**TABLE 3:** Clinical outcomes and survival analysis by cut‐off using median TPM value of CXCL13

Exploratory dataset ‐Access kit (*n* = 63)		Cutoff‐by median TPM value of CXCL13	
		Above median (*n* = 31)	Below median (*n* = 32)
PR (*n*)		14 (45.2%)	3 (9.4%)
SD (*n*)		5 (16.1%)	8 (25.0%)
PD (*n*)		12 (38.7%)	21 (65.6%)
Median PFS (95% CI)	*p* = 0.006*	129 (67–468)	42 (40–102)
Median OS (95% CI)	*p* = 0.010*	NR (458‐NR)	388 (174‐NR)
Validation dataset ‐TruSeq kit (*n* = 57)		Above median (*n* = 28)	Below median (*n* = 29)
PR (*n*)		12 (42.9%)	4 (13.8%)
SD (*n*)		3 (10.7%)	7 (24.1%)
PD (*n*)		13 (46.4%)	18 (62.1%)
Median PFS (95% CI)	*p* = 0.049*	122 (63–327)	48 (44–108)
Median OS (95% CI)	*p* = 0.025*	606 (314‐NR)	243 (128–391)

*Note*: PFS and OS are described in days.

Abbreviations: NR, not reached; OS, overall survival; PD, progressive disease; PFS, progression‐free survival; PR, partial response; SD, stable disease; TPM, transcripts per kilobase million.

* *p*‐value calculated by log‐rank test.

### 3.3 Correlation of *CXCL13* with other immune‐related biomarkers

**FIGURE 2:**
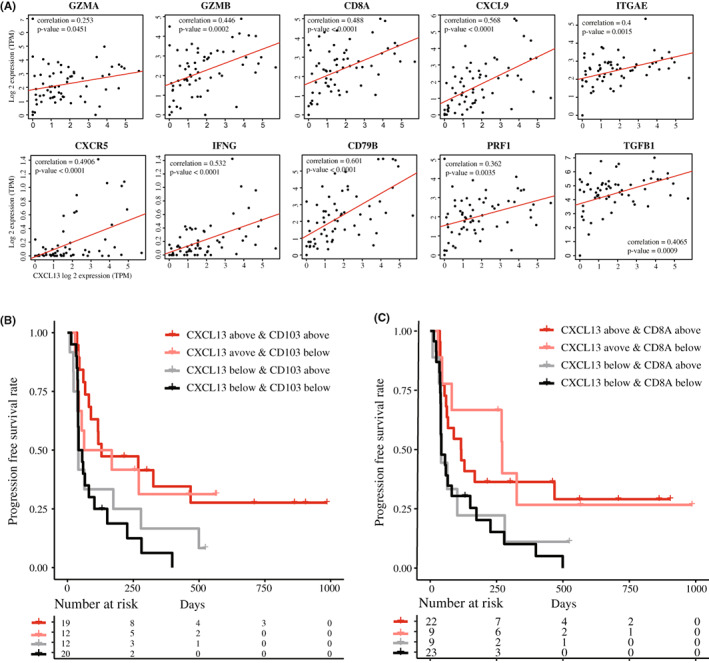
Using the exploratory dataset, (A) correlation between the expression profile of *CXCL13* and the representative genes related to the cytotoxic activity (B) Progression‐free survival analysis based on *CXCL13* and *CD103* expression, (C) *CXCL13* and *CD8A*, using median TPM as a cut‐off value from the validation dataset.

In the exploratory dataset, further investigation regarding the correlation of *CXCL13* expression with other immune‐related genes was conducted. Representative genes known to be related to the activated cytotoxic T cell,^33^ *IFNG, GZMA, GZMB, PRF1, CXCL9, CD8A, CXCR5*, and effector‐like transcriptional signature,^34^ *ITGAE/CD103*, showed a positive correlation to *CXCL13* (Figure 2A). On the contrary, the cytokine related to the poor prognosis and therapeutic resistance to ICI,^35^ *TGFB1*, showed a positive correlation to *CXCL13*. Additional analysis conducted with PD‐L1 protein expression showed that higher *CXCL13* expression was observed in samples with PD‐L1 ≥ 50% compared to PD‐L1 < 50% (*p* = 0.006, Figure S3A). Next, we tested the expression of *CXCL13* and correlation of PD‐1/PD‐L1 in a transcriptional level which showed positive trends with *PDCD1* (*p* = 0.013) and *CD274* (*p* < 0.001, Figure S3B). Based on the results of samples available for the TMB analysis (*n* = 41), the correlation between *CXCL13* and TMB was not significant (*p* = 0.054, Figure S3C). CXCL13 upregulation was favorably associated with ICI response in patients who were either PD‐L1 high or TMB high (Figure S3D). Refer to the previous report showing CD103 as an exhausted cytotoxic T‐cell marker,^36^ survival analysis using subgroups based on median TPM expression values of both genes showed that *CXCL13* seems to have a major function in deciding the response to ICI (Figure 2B). Similarly, the upregulation of genes *CXCL13* and *CD8A* showed significant PFS prolongation compared to the subgroup with both low *CXCL13* and *CD8A* (Figure 2C, *p* = 0.013).

### 3.4 Outcomes from the validation dataset

**FIGURE 3:**
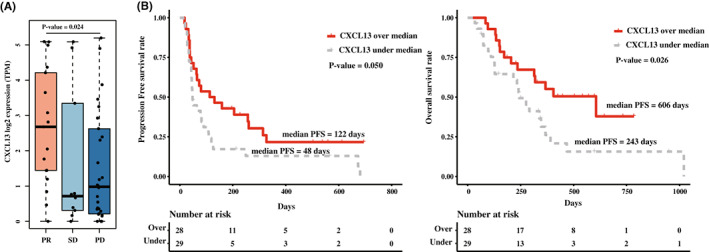
Analyses were conducted with validation dataset (*n* = 57). (A) The difference in *CXCL13* expression, is based on the best overall response to ICI. (B) Progression‐free survival and the overall survival using median *CXCL13* expression value.

In the validation dataset (*n* = 57), we conducted a similar approach, based on the response to the ICI. DEG showed significant differences in 7 genes, *CXCL13, CD8B, GZMB, IFNG, CDH6, CXCL9, and MMP1*. *CXCL13* showed a 1.76‐fold increase (*p* = 0.024) in the responder (Figure S4A and S3A). Survival analysis also showed significantly prolonged PFS (*p* = 0.050) and OS (*p* = 0.026) in high *CXCL13* expressed patients (Figure 3B, Table 3) and similar predictive values (AUC = 0.72, Figure S4B). Otherwise, similar patterns in correlation with TMB (*p* = 0.614, Figure S4C) and TLS‐related gene sets (Figure S5A) were observed. Especially, the gene related to the activated cytotoxic T‐cell and effector‐like transcriptional signature showed a positive correlation, as shown in the exploratory dataset (Figure S5B). Survival analysis conducted with subgroups categorized by the expression level of *CXCL13* and other genes of interest, *CD103*, *CD8A*, and *CXCR5*, showed that high expression of *CXCL13* is a major determinant of prolonged survival (Figure S6). We conducted additional analyses with adenocarcinoma samples from TCGA data. The results of comparing the normal samples and the tumor samples showed that *CXCL13* was significantly upregulated in tumor samples (*p* < 0.001; Figure S7A) and positively correlated to major cytolytic activity‐related genes (Figure S7B). Unlike in our dataset, OS showed no difference based on the *CXCL13* expression profile (*p* = 0.632, Figure S7C), which could be attributed to the fact that the majority of the treatment used in TCGA cohort patients consists of cytotoxic agents.

## 4 DISCUSSION

In this study, we report for the first time, a favorable association between improved clinical outcomes in ICI‐treated NSCLC and transcriptional upregulation of CXCL13 considered a surrogate marker of TLS.^17^, ^23^, ^24^, ^25^ Our outcome is consistent with the basic concept of ICI: that reinvigoration of primed exhausted TIL is important for favorable outcomes.^37^ In addition, our results are consistent with the previous report showing that PD‐1 high TIL, with a unique expression pattern of *CXCL13*, predicts favorable outcomes of PD‐1 blockade in an NSCLC cohort.^18^ Based on the strong evidence showing a correlation between *CXCL13* and the formation of TLS,^10^, ^14^, ^38^ it is a reasonable assumption that high *CXCL13* expression could influence the loco‐regional anti‐tumor immunity of tumors.^18^, ^36^ This idea is supported by the major findings that PD‐1 positive TILs with dendritic cells (DC)^hi^ TLS showed better survival compared to DC^low^ TLS in NSCLC^12^ which indicates that TLS could play a pivotal role in priming sites of immune cells with antigen‐presenting cells. In comparing the PFS of CXCL13^hi^/CD8A^hi^ patients to the PFS of CXCL13^low^/CD8^hi^ patients, we found a trend of longer PFS in the CXCL13^hi^/CD8A^hi^ group, but with a limited *p*‐value (*p* = 0.105), which could be due to the small number of patients in the subgroups (Figure 2C). Moreover, the results of the DEG analysis based on the ICI response in the exploratory dataset which showed a high *CXCR5* level in the responder also support the importance of *CXCL13*.

As a further exploratory approach, we tried to identify a direct correlation between TLS and favorable outcomes of ICI. It has been witnessed from the recent neoadjuvant study showing that post‐ICI surgical samples which showed major pathologic response had abundant TLS around the tumor.^39^ In our H&E sample, it was challenging to identify the structure due to the majority of biopsy is acquired from the core needle biopsy. Since it is difficult to identify and quantify the TLS structures in NSCLC by histological examination, we thought it is important to develop a representative biomarker that can be identified from the bulk RNA sequencing, in which *CXCL13* expression can be a potential candidate.

Despite the evidence that CXCL13 is the positive predictive biomarker for the favorable outcome of immunotherapy, there are questions regarding the causes of *CXCL13* upregulation. It is reported that *CXCL13* could be expressed by immunosuppressive cytokine, TGF‐ ß, produced by macrophages as a result of cancer‐induced chronic inflammation. which also affects the upregulation of CD103 and PD‐1 in TIL.^36^ The idea that the upregulation of *CXCL13* is a consequence of an immune‐suppressed tumor microenvironment was also shown by our results. *CXCL13* expression in the study sample showed a positive correlation to the transcription of other inhibitory genes, *PDCD1*, *CD274*, and *TGFB1*, and PD‐L1 protein expression. Hence, the underlying mechanism which triggers high *CXCL13* expression by the tumor microenvironment and how it effects the response to ICI should be carefully interpreted.

There are limitations to this study. We evaluate the CXCL13 using a different kit for library preparation in exploratory and validation cohorts. This approach was conducted to avoid the potential bias which could be derived due to the difference in the library preparation methods. Nonetheless, our findings from the exploratory cohort were reproduced in the validation cohort, which is also supported by previous reports showing no significant difference in sequencing outcomes based on the Access and TruSeq kit.^40^, ^41^ As another limitation, we conducted an analysis based on cut‐off using expression profile which has limitations in adopting to the clinical practice. For clinical utility, further investigation such as correlation with protein expression should be conducted. Last but not least, as neoadjuvant chemotherapy has become a standard treatment in potentially operable non‐small cell lung cancer,^42^ we expect that there could be more opportunities to evaluate the TLS from surgical tissue matched with clinical outcomes, which was limited to be performed with a biopsy sample.

In conclusion, we propose further clinical investigations of *CXCL13* as a predictive biomarker of ICI treatment in lung adenocarcinoma, which might reflect not only the functional status of cytotoxic T cells but also the tumor microenvironment represented by TLS. However, for clinical utility, developing a standardized method of evaluating CXCL13 expression and identifying an optimal cut‐off to recommend patients to ICI treatment along with further investigation related to the underlying mechanism should be warranted.

## AUTHOR CONTRIBUTIONS

**Sehhoon Park:** Conceptualization (equal); data curation (equal); formal analysis (equal); investigation (equal); methodology (equal); resources (equal); software (equal); supervision (equal); validation (equal); visualization (equal); writing – original draft (equal); writing – review and editing (equal). **Hongui Cha:** Data curation (equal); formal analysis (equal); methodology (equal); resources (equal); software (equal); writing – original draft (equal). **Hong Sook Kim:** Conceptualization (equal); data curation (equal); formal analysis (equal); writing – original draft (equal). **Boram Lee:** Resources (equal); software (equal); writing – original draft (equal). **Soyeon Kim:** Resources (equal). **Tae Min Kim:** Conceptualization (equal); resources (equal); writing – original draft (equal). **Hyun Ae Jung:** Resources (equal); validation (equal); writing – original draft (equal). **Jong‐Mu Sun:** Resources (equal); validation (equal); writing – review and editing (equal). **Jin Seok Ahn:** Resources (equal); validation (equal); writing – review and editing (equal). **Myung‐Ju Ahn:** Resources (equal); validation (equal); writing – review and editing (equal). **Keunchil Park:** Resources (equal); validation (equal); writing – review and editing (equal). **Woong‐Yang Park:** Resources (equal); validation (equal); writing – review and editing (equal). **Se‐Hoon Lee:** Conceptualization (equal); data curation (equal); formal analysis (equal); funding acquisition (equal); investigation (equal); methodology (equal); project administration (equal); resources (equal); software (equal); supervision (equal); validation (equal); visualization (equal); writing – original draft (equal); writing – review and editing (equal).

## FUNDING INFORMATION

This research was supported by a grant of the Korea Health Technology R&D Project through the Korea Health Industry Development Institute (KHIDI), funded by the Ministry of Health & Welfare, Republic of Korea (grant number: HR20C0025), Future Medicine 20*30 Project of the Samsung Medical Center [SMX1220091 and SMO1220071], and National Research Foundation of Korea (NRF) grant funded by the Korea government (MSIT) (No. 2020R1A2C3006535).

## CONFLICT OF INTEREST

Dr. Jin Seok Ahn reports personal fees from Amgen, personal fees from Pfizer, personal fees from AstraZeneca, personal fees from Menarini, personal fees from Roche, personal fees from Eisai, personal fees from Boehringer Ingelheim, personal fees from BMS‐Ono, personal fees from MSD, personal fees from Janssen, personal fees from Samsung Bioepis, outside the submitted work. Dr. Se‐Hoon Lee reports grants and personal fees from MSD, personal fees from Novartis, personal fees from AstraZeneca, personal fees from BMS, personal fees from Roche, outside the submitted work. Dr. Keunchil Park reports personal fees from Astellas, Astra Zeneca, AMGEN, Boehringer Ingelheim, Clovis, Eli lilly, Hanmi, KHK, Merck, MSD, Novartis, ONO, Roche, BluePrint, outside the submitted work. Dr. Sehhoon Park reports stock holding of Lunit Inc.

## Supporting information


Figure S1.

Figure S2.

Figure S3.

Figure S4.

Figure S5.

Figure S6.

Figure S7.

Figure S8.

Table S1.
Table S2.
Table S3.
Table S4.
Table S5.

## Data Availability

Data available on request due to privacy/ethical restrictions.
