# Supplementary figures and images for: Transcriptional upregulation of CXCL13 is correlated with a favorable response to immune checkpoint inhibitors in lung adenocarcinoma
Source: Cancer Med. 2022 Dec 1;12(6):7639–50. doi: 10.1002/cam4.5460 (PMC10067078; doi:10.1002/cam4.5460)

Figure S1

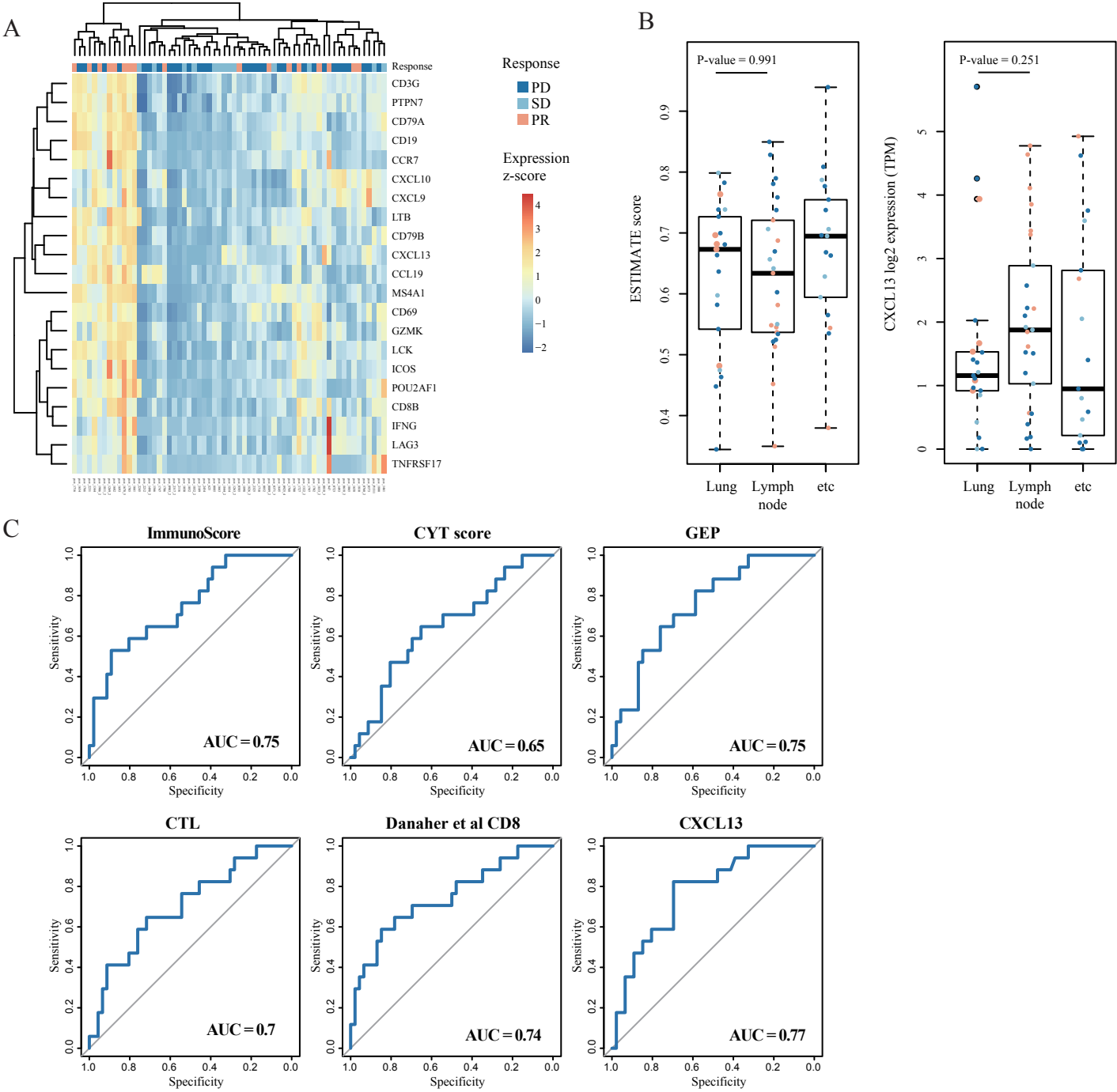

Supplement: Supplementary file 1 — Figure S1. [file CAM4-12-7639-s006.pdf]

Figure S2

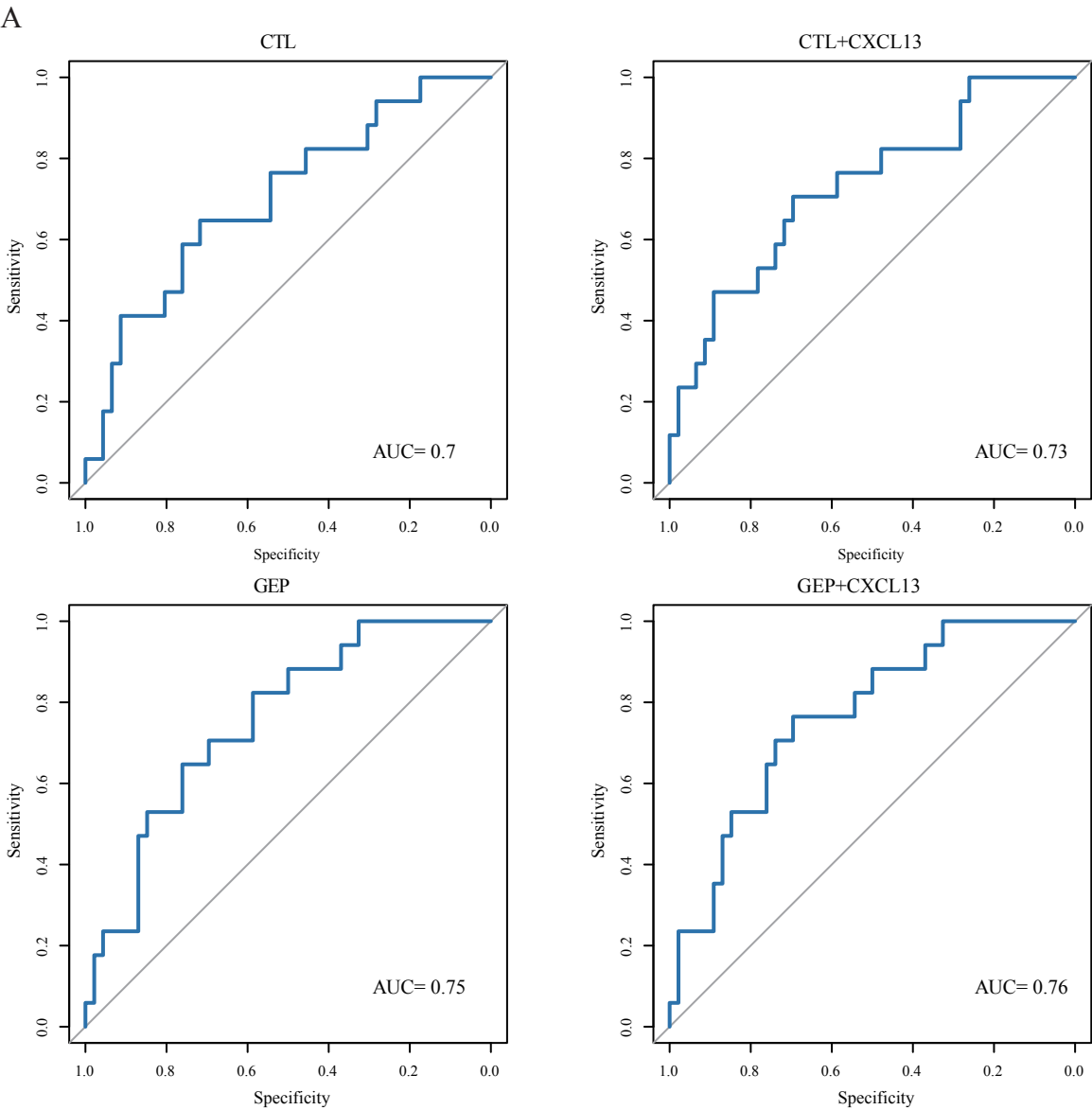

Supplement: Supplementary file 2 — Figure S2. [file CAM4-12-7639-s002.pdf]

Figure S3

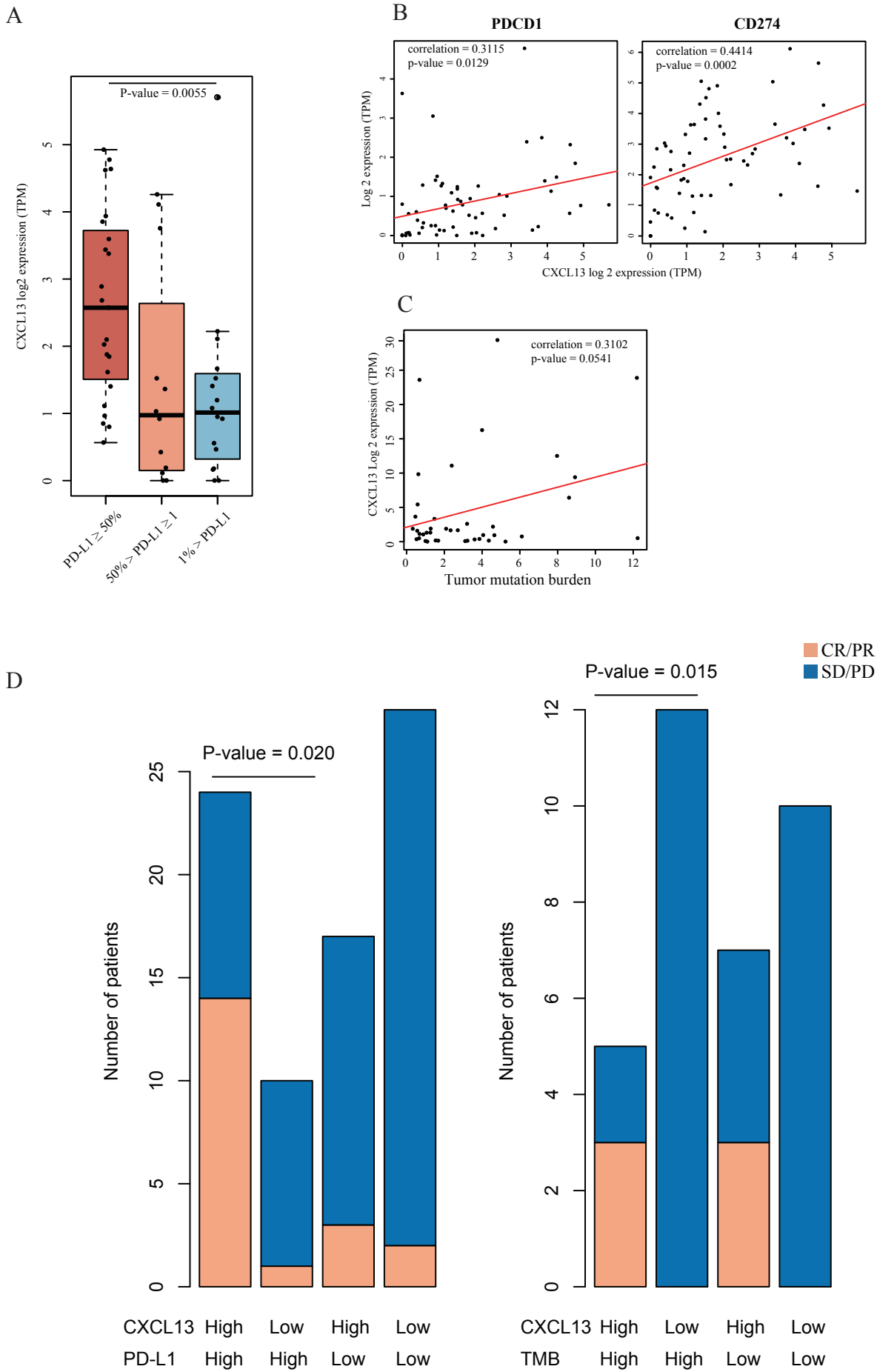

Supplement: Supplementary file 3 — Figure S3. [file CAM4-12-7639-s004.pdf]

Figure S4

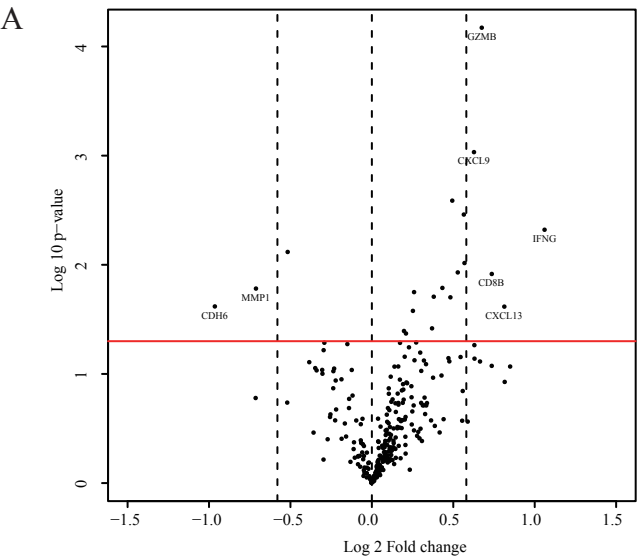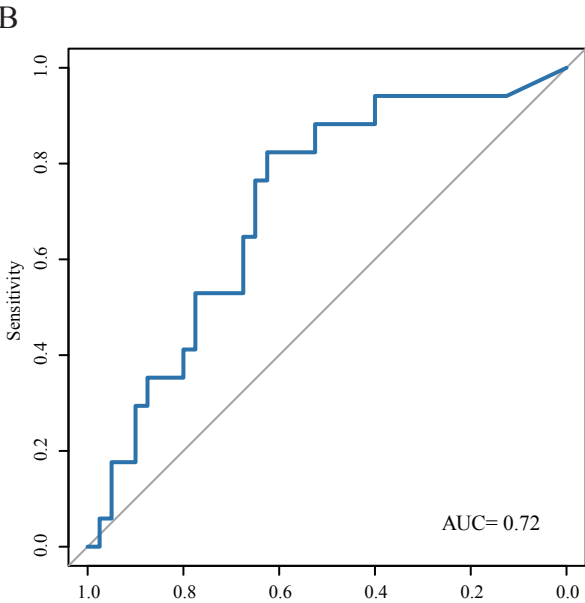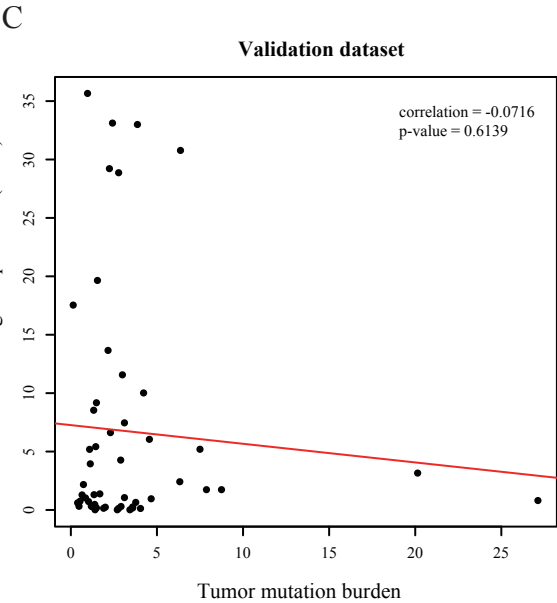

Supplement: Supplementary file 4 — Figure S4. [file CAM4-12-7639-s005.pdf]

Figure S5

A

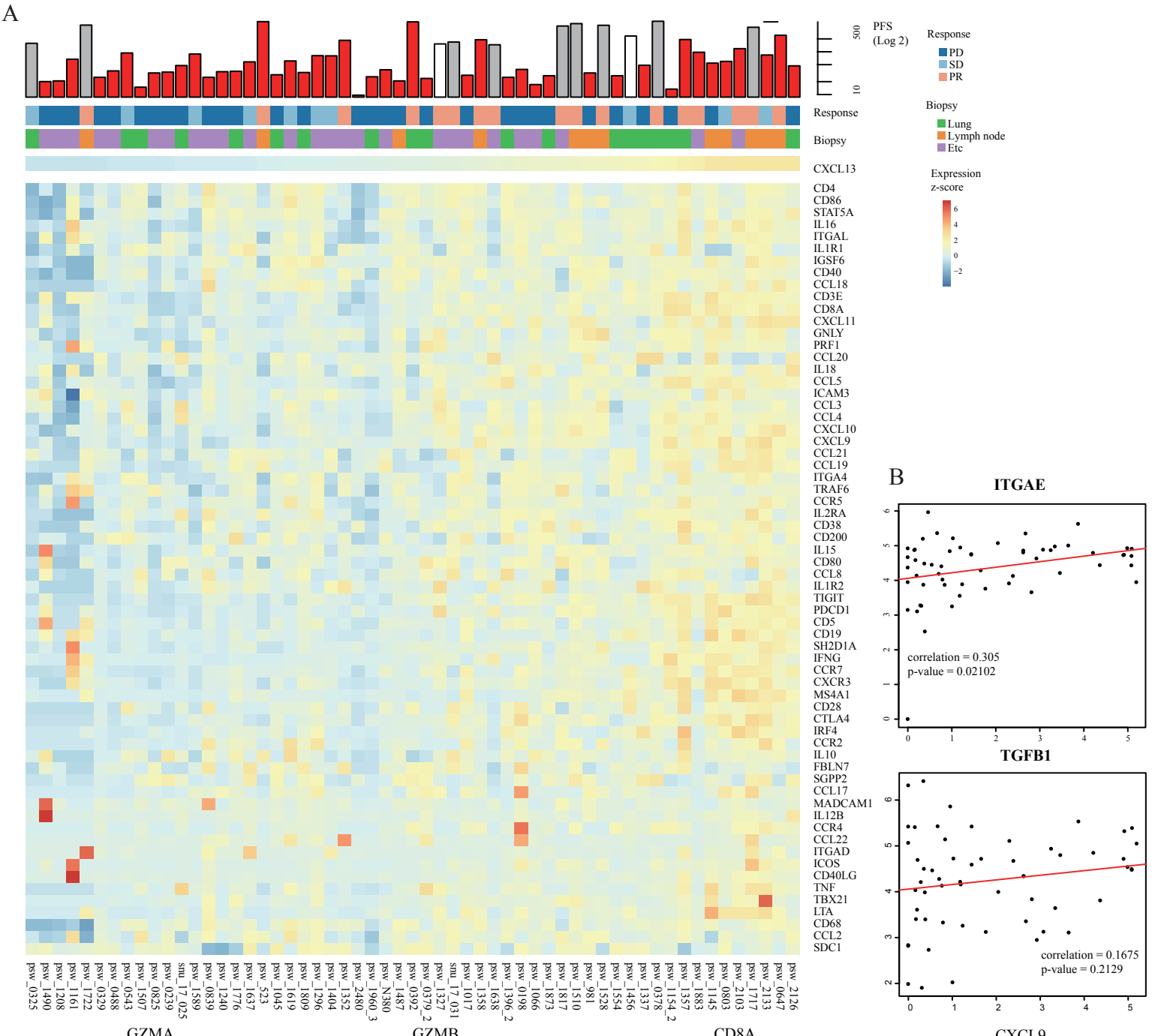

B

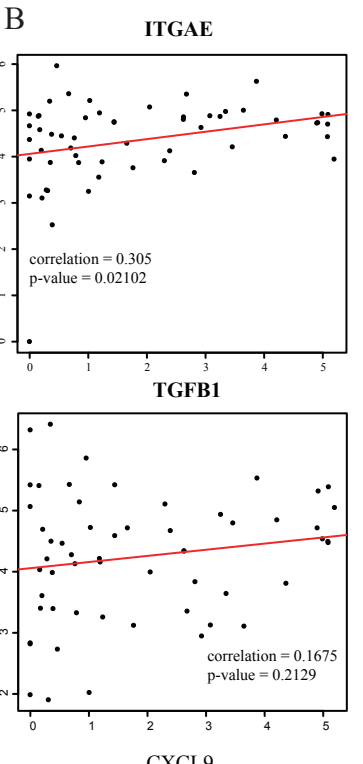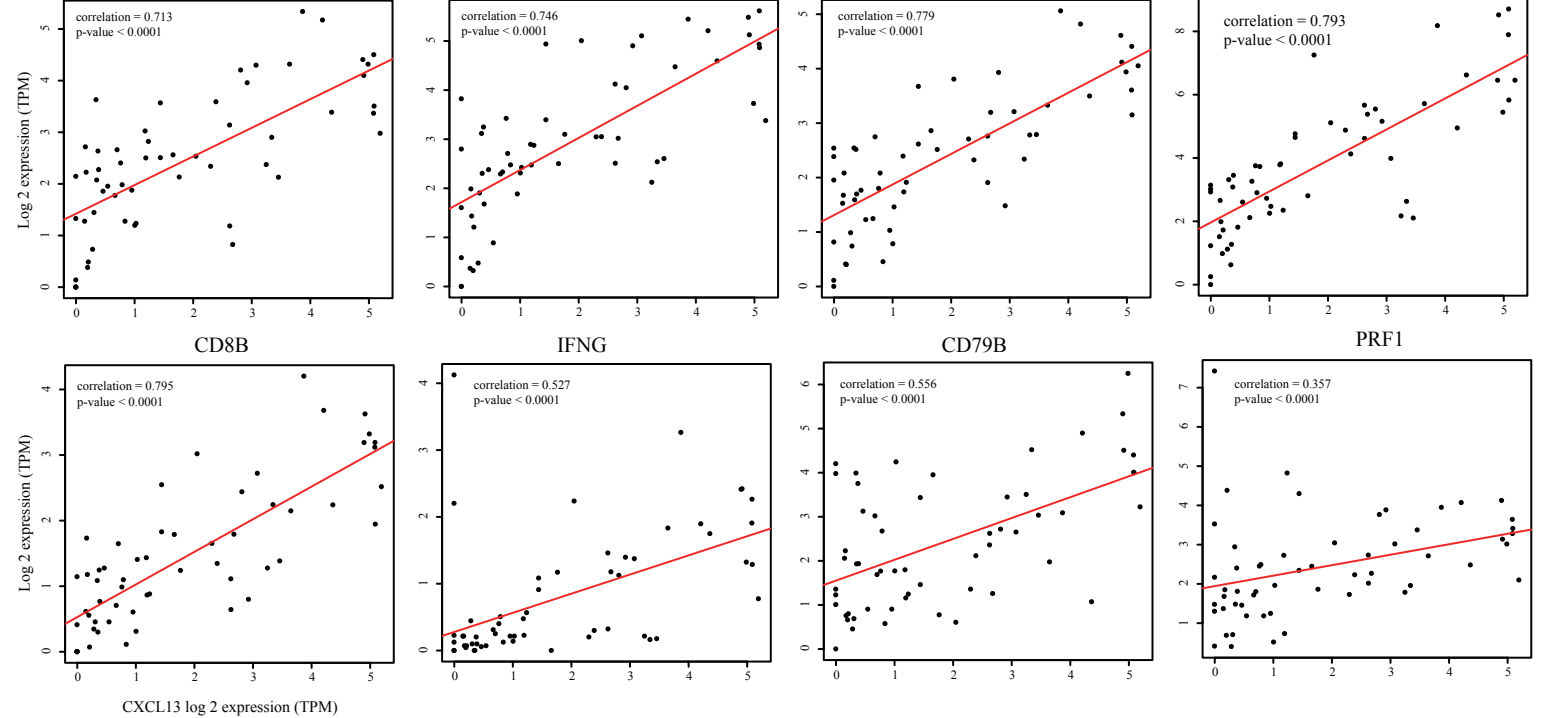

Supplement: Supplementary file 5 — Figure S5. [file CAM4-12-7639-s008.pdf]

Figure S6

A

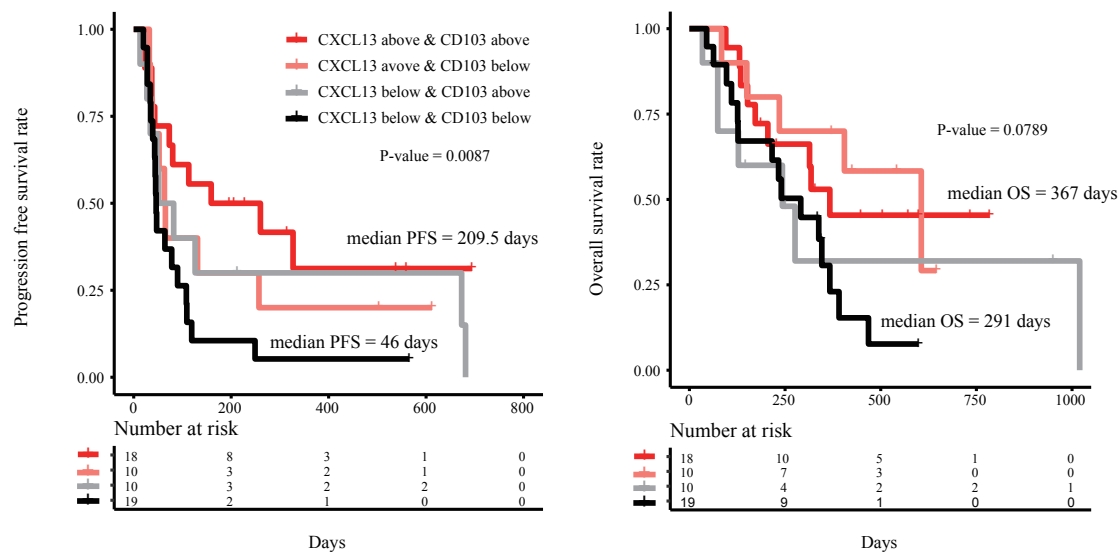

B

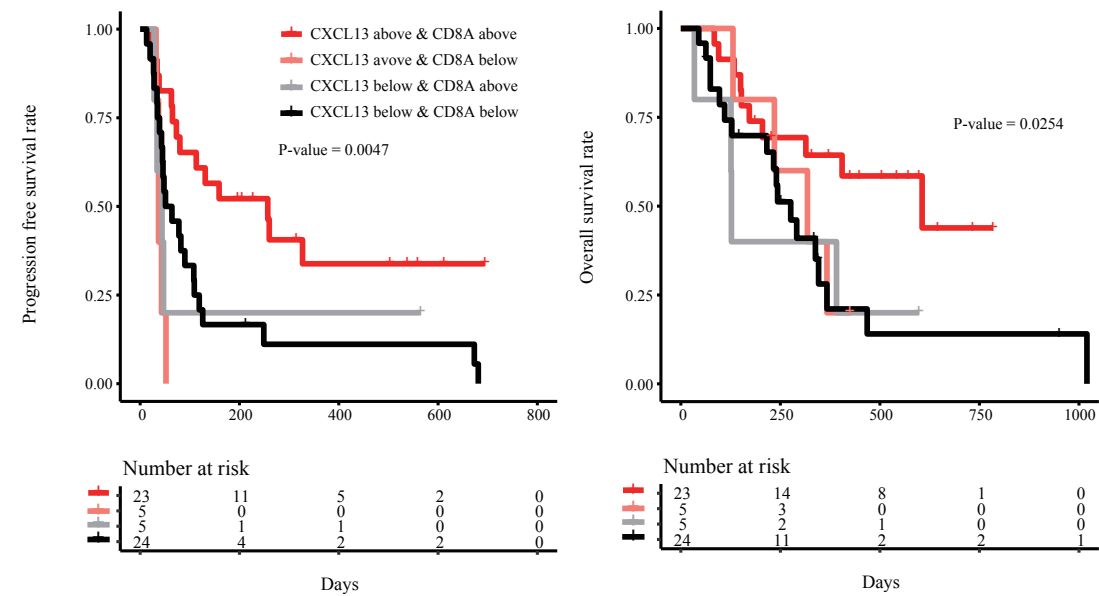

C

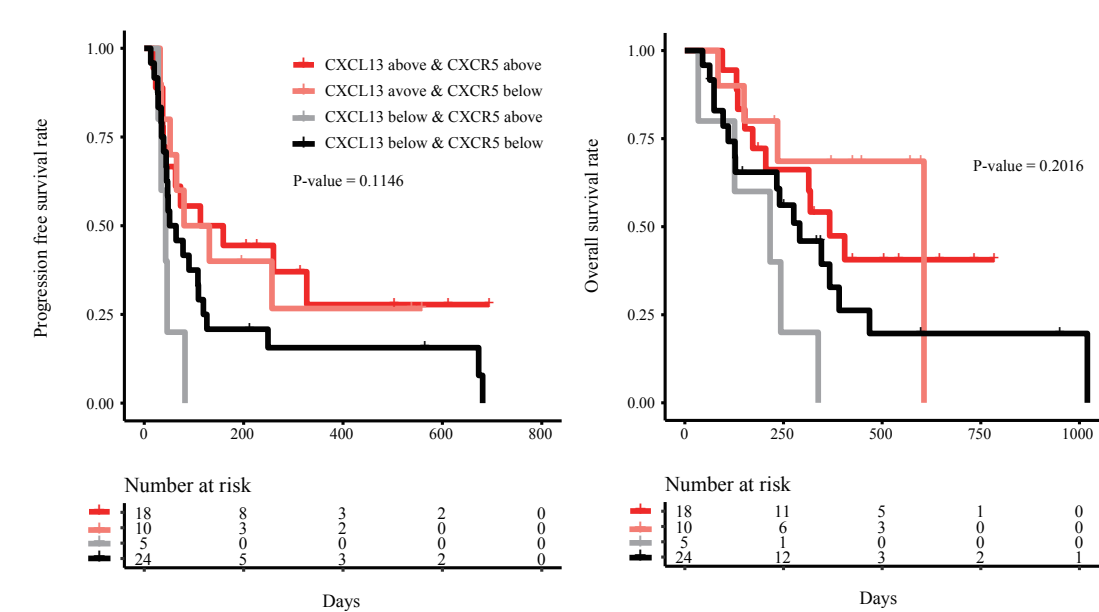

Supplement: Supplementary file 6 — Figure S6. [file CAM4-12-7639-s003.pdf]

Figure S7

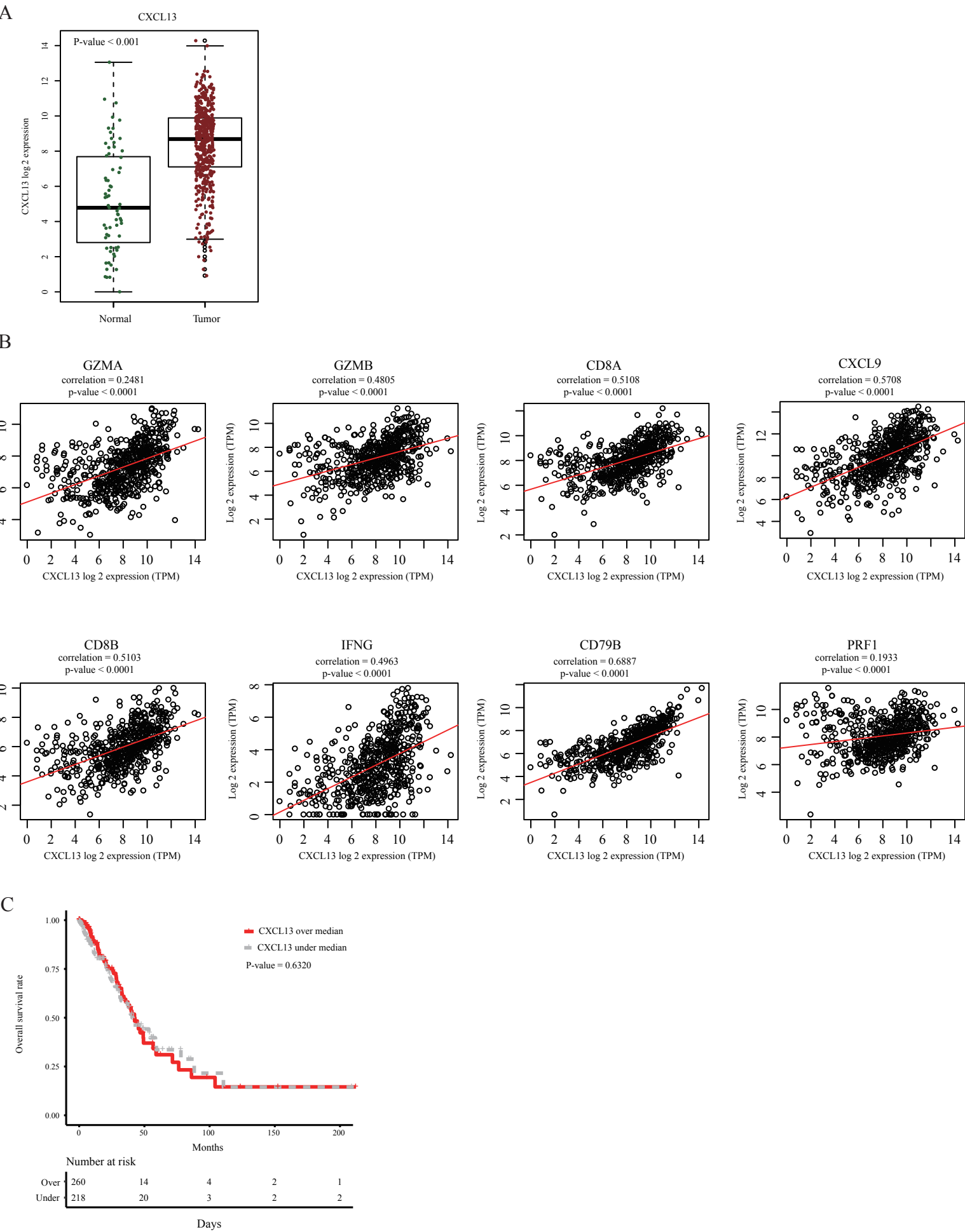

Supplement: Supplementary file 7 — Figure S7. [file CAM4-12-7639-s001.pdf]

Figure S8

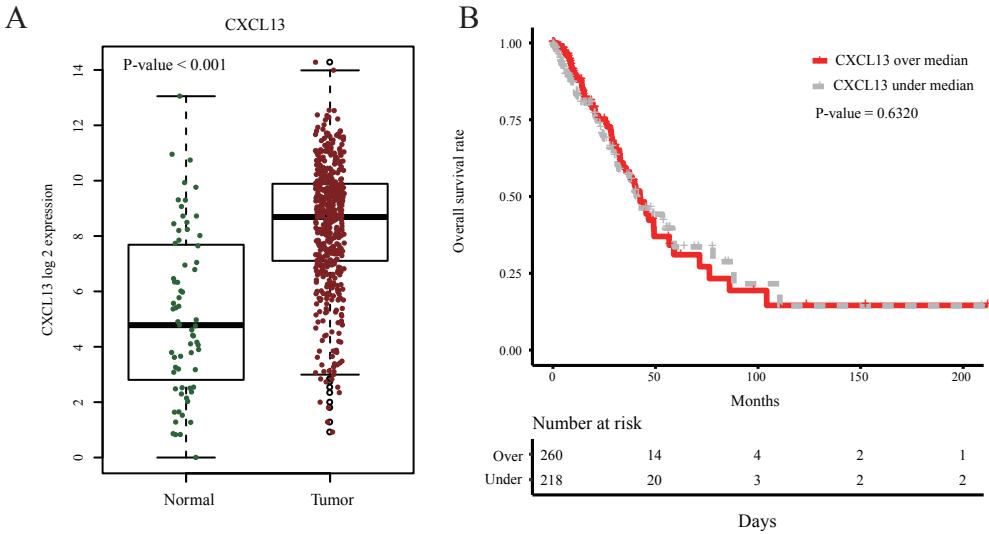

Supplement: Supplementary file 8 — Figure S8. [file CAM4-12-7639-s007.pdf]
