# Supplementary material for: Transcriptional upregulation of CXCL13 is correlated with a favorable response to immune checkpoint inhibitors in lung adenocarcinoma
Source: Cancer Med. 2022 Dec 1;12(6):7639–50. doi: 10.1002/cam4.5460 (PMC10067078; doi:10.1002/cam4.5460)
Supplement: Supplementary file 9 — Table S1. Table S2. Table S3. Table S4. Table S5. [file CAM4-12-7639-s009.docx]

**Supplementary Table 1. Raw data of study population**

| **WTS_KIT** | **Sex  M: 1, F: 2** | **Age** | **Smoking  Never: 0 Ex: 1 Current: 2** | **ECOG PS** | **Pathology** | **Study Drug** | **Previous CTx** | **Biopsy Sites** | **Previous RT** | **PD-L1 22C3 TPS** | **PFS** | **OS** | **Best Response** | **CXCL13_TPM** | **TMB_Mutect** |
| --- | --- | --- | --- | --- | --- | --- | --- | --- | --- | --- | --- | --- | --- | --- | --- |
| Access | 1 | 63 | 2 | 1 | ADC | Nivolumab | 2 | Bronchus | 0 | 1 | 45 | 103 | PD | 12.49 | 7.977678218 |
| Access | 2 | 61 | 0 | 1 | ADC | Nivolumab | 2 | Lymph node | 0 | 20 | 168 | 502 | PR | 16.25 | 4.00868408 |
| Access | 2 | 72 | 0 | 1 | ADC | Pembrolizumab | 2 | Lung | 0 | 0 | 66 | 523 | PD | 3.31 | 1.488372802 |
| Access | 1 | 72 | 0 | 1 | ADC | Pembrolizumab | 3 | Lymph node | 1 | 20 | 67 | 458 | PD | 1.87 | 2.103566893 |
| Access | 1 | 65 | 2 | 1 | ADC | Nivolumab | 2 | Lung | 1 | <1 | 38 | 545 | PD | 1.87 | 0.337364502 |
| Access | 1 | 63 | 1 | 1 | ADC | Nivolumab | 5 | Liver | 1 | 60 | 41 | 88 | PD | 1.64 | 2.341706542 |
| Access | 1 | 80 | 1 | 2 | ADC | Atezolizumab | 3 | Lung | 0 | 0 | 255 | 294 | PR | 2.17 | 4.58418823 |
| Access | 2 | 67 | 0 | 1 | ADC | Avelumab | 2 | Lymph node | 1 |  | 862 | 901 | PR | 3.63 | 0.476279297 |
| Access | 1 | 65 | 2 | 1 | ADC | Pembrolizumab | 2 | Lymph node | 0 | 99 | 301 | 337 | PR | 26.38 | 66.28220211 |
| Access | 1 | 50 | 2 | 1 | ADC | Nivolumab | 3 | Lymph node | 1 | 5 | 174 | 174 | SD | 1.04 | 0.893023681 |
| Access | 1 | 69 | 2 | 1 | ADC | Avelumab | 0 | Pleura | 0 |  | 398 | 842 | SD | 0.16 | 1.547907714 |
| Access | 1 | 83 | 0 | 1 | ADC | Nivolumab | 1 | Lymph node | 0 | <1 | 39 | 388 | PD | 0.12 | 3.234730223 |
| Access | 1 | 51 | 2 | 1 | ADC | Nivolumab | 0 | Lung | 0 |  | 986 | 1019 | PR | 1.89 | 1.289923095 |
| Access | 1 | 63 | 2 | 1 | ADC | Nivolumab | 2 | Lung | 1 | 3 | 64 | 483 | PD | 1.57 | 0.57550415 |
| Access | 1 | 61 | 0 | 1 | ADC | Pembrolizumab | 1 | Lung | 1 | 55 | 36 | 251 | PD | 3.07 |  |
| Access | 1 | 57 | 1 | 1 | ADC | Pembrolizumab | 2 | Adrenal gland | 1 | 50 | 269 | 528 | SD | 11.07 | 2.401241454 |
| Access | 2 | 44 | 0 | 1 | ADC | Nivolumab | 1 | Lung | 0 |  | 279 | 1000 | SD | 1.31 | 1.270078124 |
| Access | 2 | 63 | 0 | 1 | ADC | Pembrolizumab | 3 | Lymph node | 1 | 60 | 524 | 563 | PR | 0.48 | 0.674729004 |
| Access | 1 | 65 | 1 | 1 | ADC | Pembrolizumab | 3 | Lung | 0 | 50 | 40 | 607 | PD | 0.95 | 4.068218992 |
| Access | 1 | 73 | 1 | 1 | ADC | Nivolumab | 1 | Lymph node | 1 | 80 | 566 | 599 | PR | 2.59 | 3.214885252 |
| Access | 1 | 51 | 2 | 1 | ADC | Pembrolizumab | 3 | Heart | 0 | 80 | 461 | 500 | PR | 5.41 | 0.595349121 |
| Access | 1 | 51 | 2 | 2 | ADC | Pembrolizumab | 2 | Lung | 0 | 25 | 102 | 186 | SD | 0.34 | 0.535814209 |
| Access | 2 | 78 | 1 | 1 | ADC | Atezolizumab | 2 | Pleura | 0 | 5 | 38 | 118 | PD | 0 |  |
| Access | 1 | 60 | 2 | 1 | ADC | Pembrolizumab | 1 | Lymph node | 0 | 50 | 129 | 500 | SD | 6.40 | 8.61271728 |
| Access | 2 | 74 | 0 | 1 | ADC | Pembrolizumab | 1 | Lymph node | 0 | 50 | 562 | 595 | PR | 9.82 | 0.635039062 |
| Access | 2 | 48 | 0 | 1 | ADC | Nivolumab | 3 | Lung | 0 | 0 | 14 | 62 | PD | 0.13 | 1.686822509 |
| Access | 1 | 65 | 0 | 1 | ADC | Nivolumab | 1 | Lymph node | 1 | <1 | 35 | 563 | PD | 1.29 | 1.071628417 |
| Access | 2 | 49 | 0 | 2 | ADC | Nivolumab | 6 | Lung | 0 | 0 | 227 | 552 | PR | 1.11 | 0.694573974 |
| Access | 1 | 56 | 2 | 1 | ADC | Nivolumab | 4 | Liver | 0 |  | 40 | 74 | PD | 0.5 | 12.24434692 |
| Access | 1 | 59 | 1 | 1 | ADC | Nivolumab | 1 | Lymph node | 1 | 99 | 709 | 742 | PR | 23.87 | 12.20465698 |
| Access | 1 | 74 | 1 | 2 | ADC | Pembrolizumab | 2 | Liver | 1 | 30 | 23 | 25 | PD | 0.08 | 1.012093505 |
| Access | 2 | 42 | 0 | 1 | ADC | Pembrolizumab | 3 | Lymph node | 1 | 80 | 29 | 336 | PD | 4.95 |  |
| Access | 1 | 60 | 2 | 1 | ADC | Atezolizumab | 1 | Lung | 0 | 0 | 131 | 222 | SD | 0.00 |  |
| Access | 2 | 85 | 0 | 1 | ADC | Pembrolizumab | 0 | Lung | 0 | 60 | 215 | 215 | PR | 14.3 |  |
| Access | 2 | 63 | 0 | 1 | ADC | Nivolumab | 1 | Bronchus | 0 |  | 271 | 362 | SD | 3.14 |  |
| Access | 2 | 71 | 0 | 1 | ADC | Atezolizumab | 1 | Lung | 0 | 0 | 40 | 105 | PD | 0 | 5.248294424 |
| Access | 2 | 69 | 0 | 1 | ADC | Atezolizumab | 0 | Lung | 0 | 0 | 22 | 36 | PD | 1.65 | 2.722377321 |
| Access | 1 | 42 | 0 | 1 | ADC | Avelumab | 1 | Trachea | 0 |  | 81 | 435 | PD | 6.03 |  |
| Access | 1 | 40 | 0 | 1 | ADC | Nivolumab | 8 | Pleura | 1 | 0 | 42 | 138 | PD | 0.93 | 4.658913766 |
| Access | 1 | 81 | 2 | 1 | ADC | Durvalumab | 1 | Lymph node | 0 | 80 | 904 | 937 | PR | 9.38 | 8.930236811 |
| Access | 1 | 62 | 2 | 1 | ADC | Nivolumab | 2 | Bronchus | 0 | 0 | 151 | 392 | SD | 0.38 | 3.869769285 |
| Access | 1 | 57 | 2 | 1 | ADC | Pembrolizumab | 1 | Small intestine | 1 | 100 | 468 | 829 | PR | 29.38 | 4.822327878 |
| Access | 1 | 55 | 2 | 2 | ADC | Atezolizumab | 3 | Lymph node | 0 | 10 | 33 | 58 | PD | 0 | 1.111318359 |
| Access | 2 | 59 | 0 | 1 | ADC | Pembrolizumab | 1 | Lymph node | 1 | 95 | 326 | 570 | PR | 2.06 |  |
| Access | 1 | 50 | 2 | 1 | ADC | Pembrolizumab | 2 | Lung | 1 | 10 | 41 | 74 | PD | 18.13 |  |
| Access | 1 | 65 | 1 | 1 | ADC | Nivolumab | 2 | Lung | 0 | 0 | 499 | 542 | PR | 0.89 |  |
| Access | 1 | 68 | 1 | 1 | ADC | Pembrolizumab | 2 | Lung | 0 | 80 | 9 | 154 | PD | 1.16 |  |
| Access | 2 | 70 | 0 | 1 | ADC | Nivolumab | 3 | Lung | 0 | 0 | 38 | 195 | PD | 51.1 |  |
| Access | 1 | 68 | 0 | 1 | ADC | Pembrolizumab | 2 | Lymph node | 1 | 70 | 117 | 494 | SD | 2.67 |  |
| Access | 2 | 57 | 0 | 2 | ADC | Pembrolizumab | 3 | Lymph node | 1 | 0 | 54 | 344 | PD | 3.66 |  |
| Access | 1 | 58 | 2 | 2 | ADC | Pembrolizumab | 1 | Lymph node | 1 | 60 | 63 | 174 | PD | 3.28 |  |
| Access | 1 | 47 | 2 | 1 | ADC | Durvalumab | 2 | Bronchus | 1 |  | 59 | 314 | PD | 0 |  |
| Access | 2 | 51 | 0 | 1 | ADC | Pembrolizumab | 2 | Lung | 1 | 70 | 281 | 336 | SD | 0.80 |  |
| Access | 2 | 78 | 0 | 1 | ADC | Pembrolizumab | 1 | Lung | 0 | 25 | 56 | 236 | PD | 0.89 |  |
| Access | 1 | 52 | 2 | 1 | ADC | Nivolumab | 2 | Lymph node | 0 |  | 43 | 770 | PD | 1.84 |  |
| Access | 2 | 70 | 0 | 1 | ADC | Avelumab | 1 | Skin | 0 |  | 40 | 114 | PD | 0.07 | 3.115660399 |
| Access | 2 | 58 | 0 | 1 | ADC | Atezolizumab | 1 | Lymph node | 0 |  | 37 | 236 | PD | 0.31 | 3.611784666 |
| Access | 1 | 65 | 1 | 1 | ADC | Atezolizumab | 1 | Lymph node | 0 | 1 | 42 | 264 | PD | 0.14 | 4.365893552 |
| Access | 2 | 52 | 0 | 1 | ADC | Pembrolizumab | 1 | Thyroid gland | 0 | 70 | 89 | 482 | PD | 23.58 | 0.694573974 |
| Access | 1 | 59 | 2 | 1 | ADC | Avelumab | 2 | Brain | 0 | 80 | 81 | 837 | SD | 0.74 | 6.112250973 |
| Access | 1 | 55 | 2 | 1 | ADC | Nivolumab | 3 | Lymph node | 0 | 80 | 116 | 742 | PR | 13.44 |  |
| Access | 2 | 40 | 0 | 1 | ADC | Pembrolizumab | 1 | Lymph node | 0 |  | 60 | 1337 | SD | 2.78 |  |
| Access | 1 | 58 | 2 | 1 | ADC | Nivolumab | 1 | Lymph node | 1 | 0 | 40 | 124 | PD | 0.47 |  |
| Tru | 1 | 63 | 1 | 1 | ADC | Pembrolizumab | 3 | Pleura | 1 | 40 | 159 | 172 | PR | 30.76 | 6.390080563 |
| Tru | 1 | 47 | 1 | 1 | ADC | Pembrolizumab | 2 | Lymph node | 1 | 90 | 113 | 328 | SD | 32.98 | 3.889614256 |
| Tru | 1 | 49 | 2 | 1 | ADC | Nivolumab | 6 | Lymph node | 1 | 0 | 681 | 950 | PR | 0.38 |  |
| Tru | 1 | 71 | 1 | 2 | ADC | Durvalumab | 4 | Skin | 1 |  | 52 | 235 | PD | 4.25 | 2.917210692 |
| Tru | 1 | 73 | 2 | 2 | ADC | Atezolizumab | 3 | Lung | 0 | <1 | 34 | 84 | PD | 3.93 | 1.1510083 |
| Tru | 1 | 65 | 1 | 1 | ADC | Pembrolizumab | 2 | Lymph node | 0 | 0 | 257 | 606 | PR | 2.4 | 6.350390621 |
| Tru | 2 | 69 | 0 | 2 | ADC | Nivolumab | 4 | Bronchus | 1 | 0 | 23 | 95 | PD | 5.17 | 1.111318359 |
| Tru | 1 | 78 | 1 | 1 | ADC | Nivolumab | 2 | Lung | 1 | <1 | 20 | 233 | PD | 0.13 | 1.924962157 |
| Tru | 1 | 81 | 1 | 1 | ADC | Pembrolizumab | 1 | Lymph node | 0 | 90 | 28 | 126 | PD | 1.27 | 0.674729004 |
| Tru | 1 | 64 | 2 | 1 | ADC | Atezolizumab | 2 | Lung | 0 |  | 109 | 344 | SD | 0.7 | 1.051783447 |
| Tru | 2 | 52 | 0 | 0 | ADC | Pembrolizumab | 3 | Pleura | 0 | 55 | 119 | 334 | SD | 0.24 | 3.611784666 |
| Tru | 1 | 59 | 2 | 1 | ADC | Pembrolizumab | 2 | Bronchus | 0 | 95 | 205 | 205 | PR | 1.72 | 8.771477046 |
| Tru | 2 | 52 | 0 | 1 | ADC | Nivolumab | 2 | Liver | 1 |  | 45 | 45 | PD | 0.16 | 1.508217773 |
| Tru | 1 | 72 | 1 | 1 | ADC | Pembrolizumab | 3 | Pleura | 0 | 90 | 82 | 243 | SD | 0.59 | 0.416744385 |
| Tru | 1 | 57 | 2 | 1 | ADC | Pembrolizumab | 1 | Stomach | 0 | 60 | 196 | 448 | PR | 3.14 | 20.16249022 |
| Tru | 1 | 37 | 0 | 1 | ADC | Nivolumab | 1 | Pleura | 0 | 5 | 38 | 405 | PD | 2.16 | 0.754108886 |
| Tru | 2 | 70 | 0 | 1 | ADC | Nivolumab | 2 | Lung | 1 | 25 | 32 | 425 | PD | 1.36 | 1.70666748 |
| Tru | 1 | 56 | 1 | 1 | ADC | Nivolumab | 2 | Lung | 0 | 30 | 37 | 318 | PD | 8.52 | 1.349458007 |
| Tru | 2 | 56 | 0 | 1 | ADC | Nivolumab | 4 | Pleura | 0 | 40 | 43 | 338 | PD | 0.15 | 3.611784666 |
| Tru | 2 | 57 | 0 | 1 | ADC | Pembrolizumab | 2 | Lung | 0 | 5 | 212 | 251 | SD | 0 | 1.42883789 |
| Tru | 1 | 57 | 2 | 1 | ADC | Atezolizumab | 3 | Adrenal gland | 0 | 60 | 538 | 571 | PR | 5.4 | 1.468527831 |
| Tru | 1 | 66 | 2 | 1 | ADC | Pembrolizumab | 3 | Lymph node | 0 | 95 | 559 | 598 | PR | 7.44 | 3.135505369 |
| Tru | 1 | 66 | 2 | 1 | ADC | Avelumab | 0 | Lung | 0 | 10 | 37 | 367 | PD | 5.18 | 7.521243892 |
| Tru | 1 | 65 | 1 | 1 | ADC | Nivolumab | 1 | Lung | 0 |  | 126 | 276 | SD | 0.12 | 2.798140868 |
| Tru | 2 | 47 | 0 | 1 | ADC | Pembrolizumab | 4 | Adrenal gland | 0 | 5 | 34 | 34 | PD | 0.27 | 1.250233154 |
| Tru | 1 | 58 | 0 | 1 | ADC | Pembrolizumab | 1 | Bronchus | 0 | 95 | 131 | 371 | PR | 19.63 | 1.567752685 |
| Tru | 1 | 59 | 2 | 1 | ADC | Pembrolizumab | 3 | Lymph node | 1 | 10 | 73 | 152 | PD | 28.85 | 2.798140868 |
| Tru | 1 | 63 | 1 | 1 | ADC | Pembrolizumab | 2 | Lymph node | 0 | 60 | 327 | 504 | PR | 33.1 | 2.440931395 |
| Tru | 1 | 43 | 2 | 2 | ADC | Nivolumab | 2 | Soft tissue(Muscle) | 1 | <1 | 249 | 346 | PR | 0.79 | 27.14791991 |
| Tru | 1 | 65 | 2 | 1 | ADC | Pembrolizumab | 2 | Liver | 0 | 60 | 27 | 74 | PD | 0 | 3.4530249 |
| Tru | 1 | 48 | 1 | 1 | ADC | Pembrolizumab | 4 | Lung | 1 | 70 | 314 | 314 | SD | 9.16 | 1.508217773 |
| Tru | 2 | 34 | 0 | 1 | ADC | Nivolumab | 1 | Pleura | 0 |  | 46 | 216 | PD | 0.28 | 2.937055662 |
| Tru | 2 | 49 | 0 | 1 | ADC | Pembrolizumab | 3 | Liver | 0 | 80 | 78 | 240 | SD | 0.31 | 1.210543212 |
| Tru | 1 | 72 | 1 | 1 | ADC | Nivolumab | 1 | Lung | 0 | 10 | 39 | 63 | PD | 0.46 | 1.408992919 |
| Tru | 2 | 53 | 0 | 1 | ADC | Durvalumab | 1 | Pleura | 1 |  | 51 | 74 | PD | 1.04 | 3.135505369 |
| Tru | 1 | 67 | 2 | 1 | ADC | Pembrolizumab | 1 | Lung | 0 | 50 | 44 | 127 | PD | 0.63 | 3.790389402 |
| Tru | 1 | 59 | 0 | 0 | ADC | Atezolizumab | 1 | Lung | 0 | 0 | 63 | 425 | PD | 35.64 | 0.992248535 |
| Tru | 2 | 64 | 0 | 1 | ADC | Pembrolizumab | 3 | Lymph node | 0 | 90 | 43 | 131 | PD | 6.6 | 2.321861571 |
| Tru | 1 | 41 | 2 | 1 | ADC | Nivolumab | 6 | Pleura | 0 | 10 | 28 | 97 | PD | 0 |  |
| Tru | 1 | 51 | 1 | 1 | ADC | Pembrolizumab | 2 | Lymph node | 0 | 95 | 612 | 645 | PR | 6.03 | 4.58418823 |
| Tru | 2 | 51 | 2 | 1 | ADC | Avelumab | 2 | Lung | 0 | 90 | 694 | 733 | PR | 11.55 | 3.016435545 |
| Tru | 1 | 74 | 2 | 1 | ADC | Nivolumab | 1 | soft tissue | 0 |  | 227 | 186 | PR | 1.72 | 7.898298335 |
| Tru | 2 | 60 | 0 | 2 | ADC | Pembrolizumab | 2 | Pleura | 0 | <1 | 48 | 128 | PD | 0.11 | 4.068218992 |
| Tru | 1 | 66 | 2 | 1 | ADC | Pembrolizumab | 3 | Lung | 0 |  | 65 | 150 | PD | 10 | 4.246823728 |
| Tru | 2 | 51 | 0 | 1 | ADC | Nivolumab | 3 | Lymph node | 1 | 0 | 80 | 227 | SD | 29.2 | 2.262326659 |
| Tru | 1 | 61 | 2 | 2 | ADC | Pembrolizumab | 0 | Liver | 1 | 60 | 13 | 146 | PD | 0.94 | 4.686979512 |
| Tru | 1 | 57 | 1 | 1 | ADC | Pembrolizumab | 2 | Lung | 1 | 60 | 35 | 291 | PD | 1.01 | 0.87317871 |
| Tru | 1 | 57 | 2 | 1 | ADC | Pembrolizumab | 1 | Lung | 0 | 85 | 18 | 134 | PD | 13.64 | 2.182946776 |
| Tru | 1 | 63 | 1 | 1 | ADC | Nivolumab | 1 | Lamina Tumor | 1 | 0 | 90 | 367 | PD | 0 |  |
| Tru | 2 | 48 | 0 | 1 | ADC | Durvalumab | 0 | Lung | 0 |  | 673 | 1020 | PR | 1.29 | 1.369302978 |
| Tru | 2 | 50 | 0 | 1 | ADC | Avelumab | 0 | Lymph node | 0 | 90 | 503 | 542 | PR | 32.9 |  |
| Tru | 1 | 49 | 1 | 1 | ADC | Pembrolizumab | 6 | Lymph node | 1 | 60 | 565 | 598 | PR | 0 |  |
| Tru | 2 | 49 | 0 | 1 | ADC | Nivolumab | 2 | Lung | 0 | 70 | 260 | 784 | PR | 17.52 | 0.158759766 |
| Tru | 1 | 52 | 1 | 1 | ADC | Nivolumab | 1 | Liver | 0 | <1 | 108 | 468 | SD | 0.73 | 0.555659179 |
| Tru | 1 | 56 | 2 | 2 | ADC | Pembrolizumab | 5 | Pleura | 1 | 40 | 34 | 110 | PD | 0 | 2.718760985 |
| Tru | 2 | 33 | 0 | 1 | ADC | Pembrolizumab | 1 | Lung | 1 | 60 | 47 | 391 | PD | 0.3 | 0.496124267 |
| Tru | 2 | 48 | 0 | 1 | ADC | CA-170 | 2 | Lung | 1 |  | 64 | 64 | PD | 0.22 | 2.024187011 |

**Supplementary Table 2. Differential expressed gene analyses conducted in exploratory dataset**

| **Gene** | **P-value** | **Fold change** | **Median in PR** | **Median in SD/PD** |
| --- | --- | --- | --- | --- |
| **FN1** | 0.948296471 | 1.004028927 | 9.603398049 | 9.56486192 |
| **SEL1L3** | 0.591350817 | 1.036950232 | 5.697947438 | 5.494909268 |
| **CRTAM** | 0.076318564 | 1.644285193 | 0.816518906 | 0.496579857 |
| **UBD** | 0.248792641 | 1.201517388 | 3.761508817 | 3.130632029 |
| **RHOA** | 0.032323858 | 1.115721289 | 6.268767357 | 5.618578241 |
| **MNDA** | 0.5050033 | 1.093572176 | 2.40632714 | 2.200428277 |
| **RNASE6** | 0.216099528 | 1.252443674 | 1.856201718 | 1.482064029 |
| **LAMA4** | 0.678414735 | 0.954895279 | 4.389875704 | 4.597232596 |
| **LYZ** | 0.177205437 | 1.092851202 | 8.48110309 | 7.760528672 |
| **ITGB8** | 0.252910943 | 1.247446857 | 2.742897073 | 2.198808757 |
| **FMNL1** | 0.008417716 | 1.195487731 | 4.694119331 | 3.926530743 |
| **CYBB** | 0.082388047 | 1.128948984 | 6.278246433 | 5.561142727 |
| **HSPG2** | 0.895883255 | 0.991164912 | 6.511794764 | 6.569839878 |
| **MSN** | 0.157059613 | 1.087974805 | 5.148396404 | 4.732091568 |
| **CD3E** | 0.006986049 | 1.380306202 | 4.459205926 | 3.230591819 |
| **LRRC17** | 0.843062183 | 1.073262754 | 0.73989966 | 0.689392842 |
| **ICOS** | 0.008667533 | 2.077288617 | 1.590880895 | 0.765844901 |
| **CTSC** | 0.060698908 | 1.119962498 | 6.61141844 | 5.903249843 |
| **TFEC** | 0.011351177 | 1.35740034 | 2.734569559 | 2.01456378 |
| **WNT2B** | 0.12767783 | 0.64776286 | 0.637447604 | 0.984075568 |
| **MTA1** | 0.403567925 | 1.046271931 | 4.228711849 | 4.041694827 |
| **ARHGAP15** | 0.151285161 | 1.189772754 | 3.710041372 | 3.118277299 |
| **GZMK** | 0.01374537 | 1.715512772 | 2.448995181 | 1.427558699 |
| **COL16A1** | 0.176544359 | 0.799532885 | 2.933504595 | 3.669023064 |
| **E2F1** | 0.962684262 | 0.991208561 | 0.980397987 | 0.989093543 |
| **GNPTAB** | 0.709035491 | 1.021256707 | 4.925939478 | 4.823409674 |
| **CD14** | 0.047994196 | 1.208733657 | 3.949225699 | 3.267242272 |
| **CD19** | 0.002407077 | 2.346945207 | 2.500196346 | 1.065298132 |
| **RUNX3** | 0.04027296 | 1.415173698 | 2.064736421 | 1.458998584 |
| **FLNC** | 0.836282794 | 1.04932912 | 2.210943061 | 2.107006294 |
| **IDO1** | 0.400529746 | 1.127839634 | 2.844144839 | 2.521763515 |
| **IFI30** | 0.050239386 | 1.124014703 | 6.195559451 | 5.511991467 |
| **PXN** | 0.734582208 | 0.978407105 | 4.443194874 | 4.541253688 |
| **CCR7** | 0.011061995 | 2.158639337 | 2.05853585 | 0.953626581 |
| **MARCKSL1** | 0.539798797 | 1.085922572 | 1.724353453 | 1.587915655 |
| **PSMB9** | 0.12579838 | 1.175470655 | 4.314624887 | 3.670550913 |
| **LST1** | 0.513430849 | 1.070717666 | 3.762541552 | 3.514037052 |
| **ITGA3** | 0.444311121 | 1.066764472 | 5.526974072 | 5.181063127 |
| **CTSS** | 0.016221978 | 1.129835923 | 6.934679011 | 6.137775291 |
| **FGR** | 0.301408592 | 1.129138284 | 2.682836955 | 2.376003891 |
| **FCGR2A** | 0.677230041 | 0.967980806 | 7.006928935 | 7.23870648 |
| **CDH6** | 0.287253759 | 0.767349444 | 1.058718745 | 1.379708754 |
| **IL2RG** | 0.111271319 | 1.227604493 | 3.7818819 | 3.080700603 |
| **IL15** | 0.067138059 | 1.471273789 | 1.660273738 | 1.12846008 |
| **TRAT1** | 0.094688506 | 1.702139706 | 1.166347468 | 0.685224288 |
| **ALOX5AP** | 0.430730079 | 1.08329795 | 4.089472206 | 3.77502072 |
| **GPR171** | 0.06270036 | 1.889463746 | 1.592536105 | 0.84285084 |
| **IGJ** | 0.013066733 | 1.246844845 | 7.005512562 | 5.618592073 |
| **COL6A1** | 0.647866426 | 0.95663373 | 5.375652375 | 5.61934229 |
| **IRF1** | 0.01706484 | 1.16018924 | 4.921847033 | 4.242279504 |
| **GPNMB** | 0.169212677 | 1.088252457 | 6.162524163 | 5.662770734 |
| **ADAMDEC1** | 0.052870463 | 1.493771459 | 1.994497331 | 1.335209157 |
| **IFI16** | 0.295927999 | 1.072600473 | 5.896718627 | 5.497590926 |
| **TLR2** | 0.47423319 | 0.921774735 | 2.17304586 | 2.357458692 |
| **CTSL** | 0.31707458 | 1.060990834 | 5.868683577 | 5.53132354 |
| **FCGR2B** | 0.055276848 | 1.222904146 | 4.296671777 | 3.513498413 |
| **C3AR1** | 0.060262144 | 1.225606868 | 2.407733285 | 1.96452333 |
| **CXCL9** | 0.011943541 | 1.679268738 | 2.479084436 | 1.476288089 |
| **PLAT** | 0.971684792 | 1.006842423 | 3.001626928 | 2.981228104 |
| **IL18** | 0.054382678 | 1.240682946 | 2.970444832 | 2.394201387 |
| **CD53** | 0.00258667 | 1.227023531 | 5.8517703 | 4.769077488 |
| **PTPRC** | 0.070597296 | 1.155207198 | 6.143998117 | 5.318524787 |
| **COL8A1** | 0.907359421 | 1.025991044 | 2.490901212 | 2.427800153 |
| **HCLS1** | 0.020740216 | 1.179439135 | 5.270453271 | 4.468609794 |
| **TNC** | 0.450152729 | 1.109035008 | 4.936139284 | 4.450841722 |
| **CD84** | 0.007294297 | 1.275898323 | 3.936614646 | 3.08536705 |
| **PAK2** | 0.51063891 | 1.063814507 | 3.830082533 | 3.600329294 |
| **IGFBP5** | 0.56516572 | 0.927334442 | 3.874928222 | 4.178566055 |
| **DOCK2** | 0.009493414 | 1.192065875 | 4.933305949 | 4.138450778 |
| **CCL8** | 0.163675061 | 1.405003241 | 1.149242769 | 0.817964497 |
| **SH2B3** | 0.435178622 | 1.086325616 | 2.549421183 | 2.346829666 |
| **CTNNA1** | 0.85161366 | 0.991290167 | 7.48558095 | 7.551351966 |
| **CCL5** | 0.20527039 | 1.173524766 | 3.073995003 | 2.619454732 |
| **NCF2** | 0.274096467 | 1.084582538 | 4.240749517 | 3.910029313 |
| **LILRB4** | 0.004818905 | 1.256919184 | 4.285927096 | 3.409866881 |
| **MIF** | 0.653074262 | 0.962364707 | 4.518376242 | 4.695076836 |
| **DAPK1** | 0.842330885 | 0.986752378 | 4.131730916 | 4.187201379 |
| **DVL3** | 0.939460177 | 1.004860692 | 3.795363922 | 3.777005065 |
| **ITGA5** | 0.649237467 | 0.959587648 | 4.407692518 | 4.593319355 |
| **ITGB3** | 0.781613994 | 0.963933423 | 2.505459292 | 2.59920367 |
| **TBXAS1** | 0.156048347 | 1.087975871 | 4.210774231 | 3.870282736 |
| **FCN1** | 0.288572142 | 1.181005004 | 2.393306933 | 2.026500248 |
| **CD48** | 0.008136322 | 1.374130754 | 3.849937542 | 2.801725769 |
| **GPR18** | 0.053191494 | 2.45882385 | 1.706151416 | 0.693889241 |
| **CSF1R** | 0.108233786 | 1.130163639 | 4.836037837 | 4.279059836 |
| **SEMA3F** | 0.162431936 | 0.8256195 | 2.158884745 | 2.614866468 |
| **GIMAP4** | 0.065519968 | 1.287276015 | 3.438828692 | 2.671399647 |
| **BSG** | 0.644816334 | 1.027978389 | 4.767587944 | 4.63782896 |
| **FCGR3A** | 0.108218885 | 1.107244694 | 6.563391654 | 5.927679481 |
| **FGD1** | 0.461242819 | 1.166684682 | 1.092598188 | 0.936498272 |
| **PLEK** | 0.009358971 | 1.280799625 | 3.567640063 | 2.785478691 |
| **BTN3A3** | 0.193524107 | 1.097104861 | 5.507669224 | 5.020184872 |
| **ITK** | 0.010362013 | 1.421906625 | 3.972118275 | 2.793515555 |
| **FCER1G** | 0.517012613 | 1.063153403 | 5.019613385 | 4.721438479 |
| **PLAC8** | 0.118844366 | 1.178743283 | 4.66629295 | 3.958701626 |
| **TPM2** | 0.492345626 | 0.950685914 | 5.590608085 | 5.880604732 |
| **IGFBP4** | 0.455736097 | 0.906165457 | 3.37426598 | 3.723675357 |
| **GNLY** | 0.617537815 | 1.080088756 | 2.537895216 | 2.349709876 |
| **SP140** | 0.012902847 | 1.263931793 | 4.073972398 | 3.223253361 |
| **NKG7** | 0.082926248 | 1.384932224 | 1.747750153 | 1.261975224 |
| **ICAM1** | 0.144304278 | 1.111728953 | 5.468056098 | 4.918515508 |
| **PLCG2** | 0.221828058 | 1.139544906 | 4.10885149 | 3.605695106 |
| **RND3** | 0.793404632 | 0.97668514 | 3.935688416 | 4.029638881 |
| **VNN2** | 0.725749739 | 1.056814178 | 2.145721276 | 2.030367609 |
| **POU2AF1** | 0.011257249 | 2.070129968 | 1.586833982 | 0.766538336 |
| **TIMP1** | 0.987132544 | 0.998600903 | 6.005457486 | 6.013871476 |
| **EVI2B** | 0.145827625 | 1.155600408 | 4.897507833 | 4.238063434 |
| **TNFRSF1B** | 0.202455267 | 1.152183031 | 2.929214666 | 2.542317139 |
| **FOXM1** | 0.055926815 | 1.289955831 | 2.650288681 | 2.054557696 |
| **NPC2** | 0.699025001 | 1.028201251 | 7.344713926 | 7.143264922 |
| **RASSF2** | 0.249278162 | 1.190574481 | 1.49315801 | 1.254149181 |
| **ZBED2** | 0.243908729 | 3.615307955 | 0.313995815 | 0.086851748 |
| **PIK3CG** | 0.064241124 | 1.278066717 | 2.731498761 | 2.137211403 |
| **ISG20** | 0.189228698 | 1.152975175 | 3.650430116 | 3.166096023 |
| **THBS1** | 0.433292134 | 0.928880792 | 4.963165691 | 5.343167533 |
| **SPARC** | 0.460106647 | 0.93976374 | 7.352907129 | 7.824208165 |
| **CCL18** | 0.373676019 | 1.162039888 | 3.140124501 | 2.702251904 |
| **MMP1** | 0.979109349 | 1.007786527 | 1.841784394 | 1.827554094 |
| **LAPTM5** | 0.030368018 | 1.184317022 | 5.263202646 | 4.444082578 |
| **CCL13** | 0.086915395 | 1.56988689 | 2.215473455 | 1.411231261 |
| **ITGA4** | 0.26710015 | 1.09505684 | 4.076820856 | 3.722930817 |
| **CORO1A** | 0.001487137 | 1.242483731 | 5.397902918 | 4.344445551 |
| **RASSF4** | 0.010916913 | 1.248147285 | 3.765211807 | 3.016640625 |
| **CD44** | 0.407350838 | 1.043949646 | 7.452122127 | 7.13839231 |
| **COL3A1** | 0.247299518 | 0.888898683 | 6.95757693 | 7.827187805 |
| **FLI1** | 0.223997282 | 1.146953081 | 3.599400221 | 3.138227955 |
| **CXCL10** | 0.005184628 | 1.548979535 | 3.129181906 | 2.020157035 |
| **CD59** | 0.816825742 | 1.014123521 | 7.428620837 | 7.32516373 |
| **CCL2** | 0.282358271 | 1.120502389 | 4.784580817 | 4.270031785 |
| **CYTIP** | 0.088210135 | 1.223715863 | 3.823878589 | 3.124809201 |
| **CECR1** | 0.036057075 | 1.188817052 | 4.38160093 | 3.685681428 |
| **CXCR6** | 0.078896021 | 1.850435032 | 1.449377099 | 0.783262894 |
| **LCK** | 0.0023352 | 1.700744104 | 2.444369579 | 1.437235368 |
| **CXCL11** | 0.084986399 | 1.573006539 | 1.410097693 | 0.896434731 |
| **LAMP3** | 0.403798672 | 1.122412222 | 3.532131999 | 3.146911563 |
| **TNFRSF17** | 0.048417825 | 2.07401928 | 1.073100856 | 0.517401582 |
| **EPHB4** | 0.962097633 | 1.004406577 | 3.993174269 | 3.97565524 |
| **LAMB1** | 0.456782536 | 1.06878134 | 5.632220351 | 5.269759248 |
| **ITGB2** | 0.048564583 | 1.147851994 | 5.890896054 | 5.132104213 |
| **EPHA2** | 0.546175412 | 1.061420217 | 4.123793728 | 3.885165991 |
| **CD3G** | 0.00085474 | 1.511756066 | 3.863832875 | 2.555857364 |
| **IGFBP3** | 0.896136955 | 0.983059752 | 3.775059026 | 3.840111468 |
| **PLAUR** | 0.506539059 | 1.07691097 | 4.17785512 | 3.879480512 |
| **TNFSF13B** | 0.251535559 | 1.13894737 | 3.220094698 | 2.827255044 |
| **SLAMF7** | 0.016767294 | 1.42239065 | 2.646833781 | 1.860834631 |
| **EFNA5** | 0.613481997 | 0.929892035 | 2.382371065 | 2.561986741 |
| **FNBP1** | 0.22535532 | 1.104031755 | 4.736939106 | 4.290582299 |
| **NPL** | 0.107333154 | 1.116271984 | 4.46652761 | 4.001289717 |
| **LY86** | 0.325036071 | 1.152909262 | 1.697823995 | 1.472643208 |
| **ARHGDIA** | 0.24418485 | 1.045328661 | 6.029304441 | 5.767855282 |
| **IL7R** | 0.086499727 | 1.167083579 | 5.37726095 | 4.607434331 |
| **WARS** | 0.009471314 | 1.19321958 | 6.100691893 | 5.112799013 |
| **C1orf54** | 0.060193203 | 1.189652979 | 3.761795476 | 3.16209478 |
| **GZMB** | 0.047409109 | 1.309984572 | 2.656419306 | 2.027824879 |
| **HLA-DMA** | 0.490343242 | 1.061019177 | 5.650288355 | 5.325340464 |
| **MPP1** | 0.414145489 | 1.083565528 | 3.674483171 | 3.391103791 |
| **CXCL13** | 0.002183653 | 1.973380804 | 2.773494828 | 1.405453434 |
| **SYK** | 0.066809478 | 1.249244055 | 2.42073367 | 1.937758807 |
| **LOXL2** | 0.734001463 | 1.042359435 | 2.979902093 | 2.85880474 |
| **CD33** | 0.201307404 | 1.185520225 | 1.555353834 | 1.311958921 |
| **THBS2** | 0.677521659 | 0.93054361 | 3.074972996 | 3.304491011 |
| **GBP1** | 0.009686455 | 1.204435813 | 6.514452689 | 5.408717192 |
| **LILRB2** | 0.177358285 | 1.109577445 | 3.674853371 | 3.311939502 |
| **MMP3** | 0.59085946 | 1.35384413 | 1.095741095 | 0.809355428 |
| **EPHB2** | 0.103054384 | 1.318779358 | 2.59226128 | 1.965651998 |
| **FYB** | 0.010544941 | 1.228499445 | 5.465753825 | 4.449130075 |
| **MMP11** | 0.190615504 | 0.79139794 | 2.28526011 | 2.887624536 |
| **DSP** | 0.987227854 | 0.998435445 | 4.435646005 | 4.442596694 |
| **NCKAP1L** | 0.030156468 | 1.212593156 | 4.75363815 | 3.920225119 |
| **CORO1C** | 0.26794371 | 1.056620594 | 6.511006645 | 6.162104621 |
| **LCP1** | 0.087165358 | 1.123515568 | 6.532419715 | 5.814267199 |
| **IL10RA** | 0.129626089 | 1.126245985 | 5.186871064 | 4.605451327 |
| **SLAMF8** | 0.00059514 | 1.390152811 | 3.088713846 | 2.221852032 |
| **PLOD2** | 0.480182854 | 0.937785277 | 3.85331594 | 4.108953335 |
| **JUP** | 0.687456935 | 0.971066061 | 4.732700902 | 4.873716725 |
| **ITGB5** | 0.405248947 | 0.925995611 | 4.650544529 | 5.022210118 |
| **CD38** | 0.020361152 | 1.385192506 | 3.44875651 | 2.489730846 |
| **IL32** | 0.015992318 | 1.158445229 | 6.313664102 | 5.450118785 |
| **PTTG1** | 0.272005905 | 1.121430857 | 4.339275227 | 3.869409515 |
| **ARRB2** | 0.558990924 | 1.050787032 | 4.722128701 | 4.493897011 |
| **SELL** | 0.007129567 | 1.498365183 | 4.127233151 | 2.754490826 |
| **DVL1** | 0.43310983 | 1.081981178 | 2.719676978 | 2.513608401 |
| **NEO1** | 0.042535901 | 0.774882307 | 2.79028471 | 3.60091421 |
| **CTNNB1** | 0.856699283 | 0.992039482 | 6.848890999 | 6.903849213 |
| **ARHGAP1** | 0.894123536 | 1.009671803 | 4.206909732 | 4.166611091 |
| **CD8B** | 0.001393662 | 2.140306473 | 1.195765643 | 0.558688981 |
| **AMPD3** | 0.825741487 | 1.019178722 | 3.379852263 | 3.31625081 |
| **APOC1** | 0.832631314 | 1.012100137 | 6.774084661 | 6.69309727 |
| **GZMH** | 0.323864027 | 1.175896098 | 1.617341274 | 1.37541172 |
| **CD79A** | 0.00279274 | 1.865458717 | 3.632113871 | 1.947035246 |
| **ZYX** | 0.690491461 | 1.026624149 | 4.605582567 | 4.486142832 |
| **CD79B** | 0.00132714 | 1.8681691 | 3.420372837 | 1.83086897 |
| **MMP14** | 0.918336135 | 1.010458666 | 3.901261388 | 3.860881716 |
| **RTP4** | 0.877801419 | 0.970889525 | 0.833461427 | 0.858451354 |
| **LHFPL2** | 0.093029858 | 1.200854834 | 3.231704866 | 2.691170301 |
| **CD69** | 0.013109784 | 1.520706104 | 2.483496119 | 1.633120372 |
| **CD52** | 0.230482706 | 1.168280113 | 4.542498329 | 3.888192803 |
| **PAK1** | 0.560339544 | 1.037761236 | 4.962697277 | 4.782118568 |
| **RHOQ** | 0.709699301 | 1.034802494 | 3.524460631 | 3.405925916 |
| **CD3D** | 0.000521401 | 1.409939896 | 5.565537717 | 3.947358133 |
| **VSIG4** | 0.111470742 | 1.211312756 | 3.678437582 | 3.036736436 |
| **LCP2** | 0.012531109 | 1.142024563 | 5.585081427 | 4.890509022 |
| **HLA-DRB1** | 0.016508109 | 1.153530323 | 7.540410419 | 6.536811621 |
| **TAP1** | 0.00710219 | 1.184604373 | 6.281568653 | 5.30267218 |
| **IGSF6** | 0.01852379 | 1.23159403 | 3.73347314 | 3.031415424 |
| **LRP1** | 0.451932676 | 0.938479442 | 5.504639849 | 5.865487942 |
| **HCK** | 0.075428121 | 1.181201695 | 2.928862154 | 2.479561422 |
| **COL6A3** | 0.498841251 | 0.932789214 | 5.472268984 | 5.866565462 |
| **LAG3** | 0.009391916 | 1.808389188 | 1.661910129 | 0.919000258 |
| **B2M** | 0.313648807 | 1.029989491 | 11.12864534 | 10.80462028 |
| **EPHB3** | 0.253721411 | 0.824109475 | 1.753111006 | 2.127279275 |
| **NOTCH2** | 0.78784366 | 1.018798606 | 5.922438335 | 5.813159051 |
| **LTB** | 0.00701157 | 1.704029672 | 2.830345402 | 1.6609719 |
| **MYL6** | 0.550419736 | 1.023600483 | 7.61527469 | 7.439694312 |
| **CLEC7A** | 0.584774123 | 1.041825777 | 3.697388452 | 3.548950827 |
| **TNFRSF1A** | 0.768676327 | 1.014040253 | 6.119821073 | 6.035086924 |
| **RHOB** | 0.537392217 | 0.919397635 | 2.395279045 | 2.605269966 |
| **PIM2** | 0.063364072 | 1.285539473 | 3.33313427 | 2.592790295 |
| **CCL19** | 0.030860606 | 1.696412664 | 3.055896422 | 1.801387414 |
| **SLC1A3** | 0.705506842 | 0.961821775 | 3.276093319 | 3.40613345 |
| **MS4A1** | 0.002492419 | 1.939237091 | 4.730127977 | 2.439169506 |
| **CXCR4** | 0.176168523 | 1.148203343 | 4.667519032 | 4.065063093 |
| **HMHA1** | 0.015451686 | 1.242113214 | 4.0884633 | 3.291538367 |
| **PTPN7** | 0.000476257 | 1.510788311 | 3.300523416 | 2.184636585 |
| **FCGR1A** | 0.233013498 | 1.122250113 | 4.175449168 | 3.720604808 |
| **WNT8B** | 0.529857015 | 0.651017163 | 0.004205506 | 0.0064599 |
| **RHOG** | 0.213679615 | 1.137497528 | 2.67037506 | 2.347587571 |
| **CD8A** | 0.010178152 | 1.440723185 | 3.032826328 | 2.105072202 |
| **SAMSN1** | 0.049005798 | 1.230967531 | 3.163470764 | 2.569905935 |
| **ADAM9** | 0.148629663 | 0.918148096 | 6.145458195 | 6.69331911 |
| **FGL2** | 0.01598787 | 1.326782915 | 2.788453561 | 2.10166526 |
| **SMO** | 0.316977674 | 0.843332383 | 0.91562492 | 1.085722472 |
| **APOE** | 0.17571733 | 1.066366417 | 6.863938873 | 6.436754536 |
| **SLC7A7** | 0.028573419 | 1.166571999 | 4.330889537 | 3.712492275 |
| **SAMHD1** | 0.125249607 | 1.093226735 | 6.020914628 | 5.507471082 |
| **IFNG** | 0.002706321 | 3.626410005 | 0.443920608 | 0.122413243 |
| **IGFBP2** | 0.214906613 | 0.794846866 | 2.188277232 | 2.753080279 |
| **OSBPL3** | 0.180079162 | 1.114293841 | 4.522471446 | 4.058598621 |
| **CPVL** | 0.412936133 | 1.083174725 | 4.272245572 | 3.944188756 |
| **BIRC3** | 0.364445461 | 1.124521548 | 3.467690145 | 3.083702711 |
| **CD163** | 0.297166222 | 1.108765536 | 5.089484108 | 4.590225746 |
| **SERPINE1** | 0.559575336 | 0.917705127 | 3.258106718 | 3.550276251 |
| **EVI2A** | 0.054548116 | 1.357237734 | 2.628788491 | 1.936866641 |
| **MMP17** | 0.776971445 | 0.953691889 | 0.960442799 | 1.007078712 |
| **TAGLN** | 0.437327182 | 0.932963796 | 5.468548229 | 5.861479567 |
| **MMP19** | 0.804043237 | 0.953810633 | 1.764473763 | 1.849920416 |
| **IKZF1** | 0.019053418 | 1.413931607 | 2.870123195 | 2.029888279 |
| **LRMP** | 0.004243609 | 1.485213651 | 3.364154059 | 2.265097724 |
| **RAC1** | 0.751720835 | 1.021041641 | 6.077455905 | 5.952211604 |
| **LAIR1** | 0.00851015 | 1.237361337 | 4.115115427 | 3.325718449 |
| **CCRL2** | 0.795357279 | 1.038049291 | 1.407968066 | 1.356359546 |
| **BCL2A1** | 0.098763723 | 1.219293162 | 3.260051633 | 2.67372256 |
| **CD97** | 0.193600489 | 1.075875647 | 5.625051536 | 5.228347304 |
| **LYN** | 0.244882395 | 1.092632849 | 4.781181734 | 4.375835614 |
| **TNFAIP3** | 0.071448523 | 1.158035989 | 4.420339042 | 3.817099887 |
| **CD37** | 0.001376968 | 1.299267343 | 5.185658849 | 3.991217725 |
| **IGF2R** | 0.71715298 | 0.978719875 | 4.601296338 | 4.701341473 |
| **BTK** | 0.021303299 | 1.273467738 | 4.249684339 | 3.337096191 |
| **TCF7L2** | 0.181185468 | 0.899484489 | 3.551311332 | 3.94816295 |
| **CD86** | 0.322913073 | 1.119281605 | 2.938933209 | 2.625731716 |
| **AIM2** | 0.021382705 | 1.431335018 | 2.713801093 | 1.895992943 |
| **MYO1F** | 0.03405354 | 1.150405431 | 5.83077292 | 5.068450447 |
| **NID1** | 0.608621353 | 0.925268762 | 2.40625203 | 2.600597934 |
| **MERTK** | 0.943832773 | 1.006541822 | 3.594660533 | 3.571297739 |
| **WIPF1** | 0.474693509 | 1.074847563 | 4.450682313 | 4.140756761 |
| **SELPLG** | 0.018838471 | 1.208022765 | 4.19888089 | 3.475829273 |
| **PLA2G7** | 0.052235224 | 1.205713388 | 3.830833389 | 3.177233848 |
| **COL1A2** | 0.14591095 | 0.86272849 | 8.133869479 | 9.428075664 |
| **CASP1** | 0.046024245 | 1.156657663 | 5.498220191 | 4.753541489 |
| **ADCY7** | 0.272885103 | 1.081281695 | 3.077786145 | 2.84642398 |
| **MMP2** | 0.162047723 | 0.833164777 | 5.116162587 | 6.140637158 |

**Supplementary Table 3. List of genes which satisfy the pre-defined criteria from validation dataset.**

| **Gene** | **P-value** | **Fold change** | **Median in PR** | **Median in SD/PD** |
| --- | --- | --- | --- | --- |
| **FN1** | 0.158171194 | 0.92184684 | 9.185196979 | 9.963907866 |
| **SEL1L3** | 0.470490682 | 1.0564058 | 6.188108717 | 5.85770044 |
| **CRTAM** | 0.143647761 | 1.473337281 | 0.984997521 | 0.668548563 |
| **UBD** | 0.43120888 | 1.098209324 | 5.106157853 | 4.649530596 |
| **RHOA** | 0.257559837 | 1.027526637 | 8.409469672 | 8.184186539 |
| **MNDA** | 0.81776042 | 1.032967693 | 3.649765211 | 3.533281084 |
| **RNASE6** | 0.673422115 | 0.943465596 | 2.617608109 | 2.774460586 |
| **LAMA4** | 0.212103379 | 0.859637916 | 4.335025515 | 5.042850526 |
| **LYZ** | 0.547276503 | 1.058750834 | 6.764781785 | 6.389399251 |
| **ITGB8** | 0.183487081 | 0.698073976 | 1.093734589 | 1.566788946 |
| **FMNL1** | 0.113009217 | 1.127544051 | 4.090246381 | 3.62757125 |
| **CYBB** | 0.291113269 | 1.186007752 | 3.331592825 | 2.809081829 |
| **HSPG2** | 0.112249964 | 0.879384526 | 5.126606102 | 5.829766105 |
| **MSN** | 0.572374173 | 0.943828975 | 3.956114431 | 4.191558572 |
| **CD3E** | 0.094015423 | 1.234991778 | 2.888462554 | 2.338851647 |
| **LRRC17** | 0.345448753 | 0.780806902 | 0.806683637 | 1.033141017 |
| **ICOS** | 0.273902285 | 1.504957084 | 0.472838234 | 0.314187188 |
| **CTSC** | 0.477763729 | 1.030359864 | 8.311933295 | 8.067019678 |
| **TFEC** | 0.316322324 | 1.097685078 | 2.68228429 | 2.44358272 |
| **WNT2B** | 0.166532551 | 0.610231197 | 0.382136088 | 0.626215261 |
| **MTA1** | 0.441037763 | 0.963190149 | 4.910309079 | 5.097964387 |
| **ARHGAP15** | 0.47069411 | 1.097230552 | 2.358194555 | 2.14922429 |
| **GZMK** | 0.37006866 | 1.213401772 | 1.78545572 | 1.471446443 |
| **COL16A1** | 0.099764592 | 0.81098793 | 3.734762813 | 4.605201477 |
| **E2F1** | 0.139489486 | 1.144307678 | 3.525239888 | 3.080674854 |
| **GNPTAB** | 0.766797119 | 1.022833659 | 4.220078255 | 4.125869557 |
| **CD14** | 0.565271217 | 0.967626511 | 6.229579084 | 6.437999593 |
| **CD19** | 0.344439489 | 1.335475738 | 1.324977757 | 0.992139145 |
| **RUNX3** | 0.314184939 | 1.136035583 | 2.344716355 | 2.063946226 |
| **FLNC** | 0.996595563 | 0.99847857 | 1.019376122 | 1.020929395 |
| **IDO1** | 0.648305535 | 1.070370531 | 3.898597061 | 3.642287364 |
| **IFI30** | 0.729437461 | 1.022915973 | 7.377938809 | 7.212653829 |
| **PXN** | 0.18042536 | 1.087304629 | 5.999930992 | 5.518169272 |
| **CCR7** | 0.448502654 | 1.154574901 | 1.910442043 | 1.654671378 |
| **MARCKSL1** | 0.092172604 | 0.918708753 | 5.833835196 | 6.350037677 |
| **PSMB9** | 0.789063488 | 1.01997448 | 6.07834637 | 5.959312208 |
| **LST1** | 0.521990152 | 1.053950311 | 4.779815272 | 4.535142903 |
| **ITGA3** | 0.812885581 | 0.978988319 | 6.19320941 | 6.326132079 |
| **CTSS** | 0.455292718 | 1.045648848 | 7.064360943 | 6.755959189 |
| **FGR** | 0.12425499 | 1.142867524 | 3.858830596 | 3.376446101 |
| **FCGR2A** | 0.042698392 | 1.157157343 | 5.579809547 | 4.821997268 |
| **CDH6** | 0.024123771 | 0.513010941 | 0.437712783 | 0.853223096 |
| **IL2RG** | 0.069718475 | 1.150932359 | 5.303887619 | 4.608339995 |
| **IL15** | 0.081556004 | 1.260298455 | 2.045834954 | 1.623294027 |
| **TRAT1** | 0.085729858 | 1.802853877 | 0.596345312 | 0.330778506 |
| **ALOX5AP** | 0.91970759 | 1.012214774 | 4.309163774 | 4.257163483 |
| **GPR171** | 0.640146504 | 0.913696129 | 1.331321074 | 1.457072031 |
| **IGJ** | 0.383007777 | 1.10755778 | 6.090590761 | 5.499117852 |
| **COL6A1** | 0.423958026 | 0.927527818 | 5.655288445 | 6.097163166 |
| **IRF1** | 0.205927635 | 1.071673006 | 5.627233688 | 5.250886842 |
| **GPNMB** | 0.8978394 | 1.011244839 | 6.396337933 | 6.325211946 |
| **ADAMDEC1** | 0.260372581 | 1.358131301 | 1.705103309 | 1.255477514 |
| **IFI16** | 0.348240895 | 1.067136704 | 6.845580332 | 6.414904768 |
| **TLR2** | 0.721817266 | 0.969961535 | 3.32085803 | 3.423700745 |
| **CTSL** | 0.36285988 | 1.085581492 | 7.287791059 | 6.713260232 |
| **FCGR2B** | 0.605349453 | 1.075656622 | 3.607312089 | 3.353590741 |
| **C3AR1** | 0.522382419 | 1.061347102 | 3.830996579 | 3.609560501 |
| **CXCL9** | 0.000930576 | 1.54567616 | 5.02296331 | 3.249686732 |
| **PLAT** | 0.007644636 | 0.699399818 | 4.065070374 | 5.812226809 |
| **IL18** | 0.406849666 | 0.954669976 | 4.710971875 | 4.934660139 |
| **CD53** | 0.470826004 | 1.080057483 | 4.73677571 | 4.385670009 |
| **PTPRC** | 0.194753439 | 1.243241637 | 3.133504283 | 2.520430614 |
| **COL8A1** | 0.75582015 | 1.176132905 | 0.304371968 | 0.258790454 |
| **HCLS1** | 0.900670833 | 0.993784184 | 5.7185128 | 5.754280345 |
| **TNC** | 0.512119681 | 1.08430562 | 5.437007146 | 5.014275538 |
| **CD84** | 0.038309884 | 1.292968208 | 3.258054239 | 2.519825482 |
| **PAK2** | 0.51606374 | 1.075451124 | 3.96332339 | 3.685265934 |
| **IGFBP5** | 0.988310701 | 1.00178393 | 5.037477418 | 5.028506912 |
| **DOCK2** | 0.265820303 | 1.13907033 | 2.905985094 | 2.551190228 |
| **CCL8** | 0.233564879 | 1.255512871 | 2.035068007 | 1.62090573 |
| **SH2B3** | 0.537916735 | 1.155417603 | 1.559767332 | 1.349959814 |
| **CTNNA1** | 0.975030534 | 0.998411118 | 7.292837635 | 7.304443536 |
| **CCL5** | 0.017833493 | 1.197769405 | 5.436434696 | 4.538799097 |
| **NCF2** | 0.307182315 | 1.081071803 | 4.250759313 | 3.931986111 |
| **LILRB4** | 0.764902214 | 1.03769419 | 3.384381846 | 3.261444342 |
| **MIF** | 0.526812174 | 0.979805187 | 8.840536674 | 9.022749414 |
| **DAPK1** | 0.235250176 | 0.839059317 | 3.000214299 | 3.575687962 |
| **DVL3** | 0.770620799 | 1.027209447 | 3.902960767 | 3.799576394 |
| **ITGA5** | 0.981647018 | 0.99819394 | 5.469355641 | 5.479251495 |
| **ITGB3** | 0.299913735 | 1.308338255 | 1.839610388 | 1.406066345 |
| **TBXAS1** | 0.764559421 | 0.96741443 | 3.09392376 | 3.198136874 |
| **FCN1** | 0.846734901 | 1.036539097 | 2.726780287 | 2.630658405 |
| **CD48** | 0.121902652 | 1.162837084 | 4.418497583 | 3.799756341 |
| **GPR18** | 0.412413962 | 1.23852399 | 1.286634932 | 1.038845385 |
| **CSF1R** | 0.975964067 | 1.002463556 | 4.914834123 | 4.902755911 |
| **SEMA3F** | 0.39349673 | 0.879873476 | 2.681011858 | 3.047042478 |
| **GIMAP4** | 0.957682268 | 1.010439694 | 1.453008095 | 1.437995859 |
| **BSG** | 0.866891214 | 0.990528181 | 7.638808405 | 7.71185369 |
| **FCGR3A** | 0.330881924 | 1.061239598 | 6.598247598 | 6.217490955 |
| **FGD1** | 0.487439414 | 1.106181461 | 1.516125835 | 1.370594147 |
| **PLEK** | 0.553740226 | 1.086644944 | 2.944914939 | 2.710098598 |
| **BTN3A3** | 0.586919832 | 1.050659858 | 3.855032231 | 3.669153439 |
| **ITK** | 0.054591062 | 1.548259726 | 1.61428203 | 1.042642912 |
| **FCER1G** | 0.593050195 | 1.043098814 | 6.091029804 | 5.839360302 |
| **PLAC8** | 0.604856823 | 1.083188914 | 4.003440571 | 3.695976315 |
| **TPM2** | 0.205868454 | 0.907946363 | 5.780261094 | 6.366302384 |
| **IGFBP4** | 0.285066085 | 0.892060438 | 4.716990255 | 5.287747396 |
| **GNLY** | 0.009657319 | 1.484601947 | 3.682485811 | 2.480453309 |
| **SP140** | 0.071835618 | 1.386505276 | 2.265538965 | 1.633992314 |
| **NKG7** | 0.002590392 | 1.409204051 | 4.647697407 | 3.298101082 |
| **ICAM1** | 0.815541377 | 0.979212395 | 5.436324472 | 5.551731677 |
| **PLCG2** | 0.388671537 | 1.082225772 | 3.454541921 | 3.192071388 |
| **RND3** | 0.223400588 | 1.108339891 | 4.601552121 | 4.151751784 |
| **VNN2** | 0.184565749 | 1.234403548 | 3.12526358 | 2.531800548 |
| **POU2AF1** | 0.389803545 | 1.22581444 | 2.088615636 | 1.703859547 |
| **TIMP1** | 0.289406807 | 0.954801409 | 9.096879121 | 9.527509109 |
| **EVI2B** | 0.1517993 | 1.117026906 | 4.435669824 | 3.970960591 |
| **TNFRSF1B** | 0.504943312 | 1.089403205 | 3.318478511 | 3.046143519 |
| **FOXM1** | 0.825386711 | 1.019762766 | 4.109619963 | 4.029976481 |
| **NPC2** | 0.25819486 | 0.961721018 | 9.168944419 | 9.533892106 |
| **RASSF2** | 0.797589539 | 1.025896433 | 2.981115346 | 2.905863838 |
| **ZBED2** | 0.084531618 | 1.666934654 | 1.677418795 | 1.006289473 |
| **PIK3CG** | 0.194131001 | 1.249443047 | 1.243572256 | 0.995301273 |
| **ISG20** | 0.61333563 | 1.029897065 | 4.986776989 | 4.842014952 |
| **THBS1** | 0.094567365 | 0.849527064 | 4.893681171 | 5.760477067 |
| **SPARC** | 0.169648267 | 0.908467925 | 9.285692463 | 10.22126617 |
| **CCL18** | 0.376956234 | 1.154221447 | 3.685357874 | 3.192938308 |
| **MMP1** | 0.016550526 | 0.611103851 | 2.350529041 | 3.846365945 |
| **LAPTM5** | 0.304573775 | 1.03851281 | 7.293393146 | 7.022920733 |
| **CCL13** | 0.318256215 | 1.231145095 | 2.931560477 | 2.381165703 |
| **ITGA4** | 0.194810734 | 1.19559234 | 2.810397098 | 2.350631569 |
| **CORO1A** | 0.806858836 | 1.015540881 | 5.718980342 | 5.631462453 |
| **RASSF4** | 0.698190292 | 1.032136155 | 3.950644812 | 3.827639204 |
| **CD44** | 0.712512587 | 1.02192107 | 7.20043158 | 7.04597624 |
| **COL3A1** | 0.135591632 | 0.849079202 | 6.717428217 | 7.911427108 |
| **FLI1** | 0.887840232 | 1.022241183 | 2.145374543 | 2.098697038 |
| **CXCL10** | 0.016325737 | 1.351203652 | 5.304766909 | 3.925956609 |
| **CD59** | 0.415564709 | 1.028175673 | 8.492268391 | 8.259550012 |
| **CCL2** | 0.697512435 | 1.021904498 | 6.500970728 | 6.361622581 |
| **CYTIP** | 0.075147201 | 1.199914906 | 3.601896138 | 3.001792977 |
| **CECR1** | 0.447135751 | 1.068094243 | 4.363923575 | 4.085710228 |
| **CXCR6** | 0.185302783 | 1.265280826 | 1.354129466 | 1.07022049 |
| **LCK** | 0.194844345 | 1.261440452 | 2.0950118 | 1.660809114 |
| **CXCL11** | 0.103621452 | 1.346156693 | 2.754002419 | 2.045826042 |
| **LAMP3** | 0.889776717 | 0.979161347 | 3.470659779 | 3.544522862 |
| **TNFRSF17** | 0.268902063 | 1.469960825 | 1.037820681 | 0.706019278 |
| **EPHB4** | 0.723886306 | 0.97972056 | 5.444463212 | 5.557159291 |
| **LAMB1** | 0.115148807 | 0.857980842 | 5.22908446 | 6.094640114 |
| **ITGB2** | 0.62847818 | 1.027326549 | 6.803941664 | 6.622959051 |
| **EPHA2** | 0.563616065 | 0.95049827 | 4.338665878 | 4.564622592 |
| **CD3G** | 0.077106089 | 1.585873691 | 1.186815239 | 0.748366812 |
| **IGFBP3** | 0.564001301 | 1.039064221 | 7.245357824 | 6.9729644 |
| **PLAUR** | 0.32596565 | 1.066171487 | 6.185957884 | 5.802028996 |
| **TNFSF13B** | 0.184774306 | 1.120219737 | 4.013574583 | 3.582845803 |
| **SLAMF7** | 0.16513061 | 1.252441647 | 2.877480389 | 2.29749657 |
| **EFNA5** | 0.472948254 | 1.086846437 | 3.253605982 | 2.99362069 |
| **FNBP1** | 0.040494334 | 1.147463478 | 4.413517645 | 3.846325159 |
| **NPL** | 0.58211588 | 1.063394584 | 3.289143127 | 3.093059883 |
| **LY86** | 0.807864659 | 0.969885333 | 3.05754729 | 3.152483274 |
| **ARHGDIA** | 0.941542686 | 0.997614696 | 7.982964291 | 8.002051618 |
| **IL7R** | 0.019908071 | 1.398052709 | 2.819325333 | 2.016608756 |
| **WARS** | 0.275082461 | 1.068307962 | 7.534200607 | 7.052461347 |
| **C1orf54** | 0.745609014 | 1.033575222 | 4.392403257 | 4.249718032 |
| **GZMB** | 6.74E-05 | 1.597680946 | 3.972907233 | 2.486671224 |
| **HLA-DMA** | 0.741269059 | 0.986653323 | 7.053648207 | 7.149064464 |
| **MPP1** | 0.152275788 | 1.075789604 | 4.952320231 | 4.60342823 |
| **CXCL13** | 0.024227431 | 1.758775967 | 2.656620332 | 1.510493878 |
| **SYK** | 0.970004176 | 1.005732149 | 1.543799107 | 1.535000257 |
| **LOXL2** | 0.753107985 | 1.030219683 | 4.851468535 | 4.709159236 |
| **CD33** | 0.628617349 | 1.078796686 | 2.122649009 | 1.967608019 |
| **THBS2** | 0.078192219 | 0.766719597 | 3.854920922 | 5.027810608 |
| **GBP1** | 0.283653362 | 1.074702727 | 5.412629844 | 5.036397238 |
| **LILRB2** | 0.306624276 | 1.115951708 | 2.790787881 | 2.500814201 |
| **MMP3** | 0.610285274 | 0.814689616 | 1.051210669 | 1.290320446 |
| **EPHB2** | 0.616315341 | 1.092586403 | 2.384368161 | 2.182315425 |
| **FYB** | 0.221135034 | 1.188835244 | 3.48405144 | 2.930642793 |
| **MMP11** | 0.092978222 | 0.79106538 | 3.560682849 | 4.501123344 |
| **DSP** | 0.593705376 | 1.130946286 | 1.99777994 | 1.766467571 |
| **NCKAP1L** | 0.063907587 | 1.22951738 | 3.274072145 | 2.662892123 |
| **CORO1C** | 0.411250045 | 1.059564305 | 6.299888206 | 5.945734654 |
| **LCP1** | 0.08563457 | 1.119825153 | 6.523857783 | 5.825782502 |
| **IL10RA** | 0.264949386 | 1.131818005 | 3.586533102 | 3.168824923 |
| **SLAMF8** | 0.16398637 | 1.185032655 | 3.191512407 | 2.693185199 |
| **PLOD2** | 0.026498619 | 1.191856814 | 6.649914384 | 5.579457454 |
| **JUP** | 0.053375579 | 0.901701216 | 5.820682326 | 6.455222888 |
| **ITGB5** | 0.861437662 | 1.009656468 | 6.377949868 | 6.316950438 |
| **CD38** | 0.108467069 | 1.298602453 | 2.21500504 | 1.705683703 |
| **IL32** | 0.925342093 | 1.006169873 | 6.313170401 | 6.274457796 |
| **PTTG1** | 0.191477819 | 1.12076455 | 5.22412615 | 4.661216444 |
| **ARRB2** | 0.852111583 | 1.015758024 | 3.843622708 | 3.783994434 |
| **SELL** | 0.262385972 | 1.151246514 | 3.653744334 | 3.1737289 |
| **DVL1** | 0.561950022 | 1.034803566 | 4.949743408 | 4.783268602 |
| **NEO1** | 0.088610127 | 0.786460826 | 2.480650074 | 3.15419407 |
| **CTNNB1** | 0.928329208 | 0.994682832 | 6.761523536 | 6.797667879 |
| **ARHGAP1** | 0.430213057 | 0.956332358 | 5.086558794 | 5.318819083 |
| **CD8B** | 0.012161871 | 1.667324844 | 2.016361566 | 1.209339364 |
| **AMPD3** | 0.129891134 | 1.184164558 | 2.946378099 | 2.488149202 |
| **APOC1** | 0.606280048 | 0.964589811 | 7.602502171 | 7.88159079 |
| **GZMH** | 0.011770515 | 1.442615705 | 3.198108719 | 2.216881951 |
| **CD79A** | 0.267169596 | 1.28771895 | 2.545227187 | 1.976539358 |
| **ZYX** | 0.525192834 | 0.960651484 | 5.354703575 | 5.574033521 |
| **CD79B** | 0.588453154 | 1.108123789 | 2.606719401 | 2.352372025 |
| **MMP14** | 0.986058403 | 1.001823368 | 5.654392644 | 5.644101369 |
| **RTP4** | 0.670463931 | 1.100379036 | 1.265795448 | 1.150326757 |
| **LHFPL2** | 0.354137612 | 1.077768142 | 4.069643263 | 3.775991425 |
| **CD69** | 0.075534976 | 1.249036281 | 2.765176966 | 2.213848395 |
| **CD52** | 0.051617064 | 1.208550873 | 6.059525577 | 5.013877127 |
| **PAK1** | 0.654940162 | 1.026785019 | 5.641345489 | 5.494183672 |
| **RHOQ** | 0.410450496 | 1.097002759 | 3.474695582 | 3.167444707 |
| **CD3D** | 0.019652663 | 1.302414175 | 3.794730657 | 2.913612835 |
| **VSIG4** | 0.990115018 | 0.998776389 | 4.643040419 | 4.648728655 |
| **LCP2** | 0.052030552 | 1.128233155 | 4.719633976 | 4.183208014 |
| **HLA-DRB1** | 0.458183942 | 0.967568004 | 7.767339506 | 8.02769363 |
| **TAP1** | 0.236705615 | 1.066513483 | 6.155209616 | 5.771337833 |
| **IGSF6** | 0.719649933 | 1.044335141 | 3.790981394 | 3.63004293 |
| **LRP1** | 0.488928665 | 0.92599367 | 4.530275654 | 4.892339765 |
| **HCK** | 0.248352979 | 1.155102935 | 2.916727262 | 2.525079951 |
| **COL6A3** | 0.375379104 | 0.896827971 | 4.941154681 | 5.50959029 |
| **LAG3** | 0.076903576 | 1.39176837 | 1.916365049 | 1.376928151 |
| **B2M** | 0.42017025 | 1.029710653 | 12.20719819 | 11.85497902 |
| **EPHB3** | 0.248489339 | 0.837548828 | 2.232146419 | 2.665094074 |
| **NOTCH2** | 0.685119952 | 1.035323866 | 4.063898758 | 3.925243966 |
| **LTB** | 0.488652749 | 1.079589861 | 4.600185409 | 4.261049105 |
| **MYL6** | 0.657312558 | 0.98591211 | 10.44929258 | 10.59860455 |
| **CLEC7A** | 0.576670124 | 1.05851475 | 4.311233726 | 4.072908503 |
| **TNFRSF1A** | 0.10621876 | 1.08444864 | 7.243399709 | 6.679338647 |
| **RHOB** | 0.911270593 | 0.993437107 | 5.822077073 | 5.860539165 |
| **PIM2** | 0.184356453 | 1.141342832 | 3.783279309 | 3.314761528 |
| **CCL19** | 0.340063391 | 1.219369243 | 4.240559898 | 3.47766677 |
| **SLC1A3** | 0.456972232 | 1.12205947 | 2.919465427 | 2.601881187 |
| **MS4A1** | 0.118561976 | 1.761562504 | 2.291520846 | 1.300845607 |
| **CXCR4** | 0.171426032 | 1.093071188 | 5.285367394 | 4.835336851 |
| **HMHA1** | 0.773689537 | 1.020782287 | 4.85768339 | 4.758784956 |
| **PTPN7** | 0.17235822 | 1.145829403 | 3.34149821 | 2.916226622 |
| **FCGR1A** | 0.851530493 | 1.023969278 | 3.291968273 | 3.21490922 |
| **WNT8B** | NaN | NaN | 0 | 0 |
| **RHOG** | 0.966529061 | 0.998817783 | 5.651403996 | 5.658093089 |
| **CD8A** | 0.003472797 | 1.480367728 | 3.045042538 | 2.056950094 |
| **SAMSN1** | 0.057156882 | 1.172000109 | 3.460874299 | 2.952964145 |
| **ADAM9** | 0.315582656 | 1.115301624 | 5.627950745 | 5.046124406 |
| **FGL2** | 0.119932657 | 1.159482632 | 3.7913287 | 3.269845183 |
| **SMO** | 0.397600429 | 0.829122082 | 1.168425749 | 1.409232457 |
| **APOE** | 0.267567211 | 0.9364901 | 7.43500652 | 7.939225966 |
| **SLC7A7** | 0.40841869 | 1.074916223 | 4.809962857 | 4.474732778 |
| **SAMHD1** | 0.64124084 | 1.025674193 | 5.137436472 | 5.008838581 |
| **IFNG** | 0.004785331 | 2.087311693 | 1.27496747 | 0.61081796 |
| **IGFBP2** | 0.060701235 | 0.815045413 | 5.324994866 | 6.533371981 |
| **OSBPL3** | 0.640021978 | 1.04763441 | 3.680225105 | 3.512890631 |
| **CPVL** | 0.951080137 | 1.004719375 | 5.536924312 | 5.510916232 |
| **BIRC3** | 0.630281573 | 1.054121549 | 3.483199769 | 3.304362551 |
| **CD163** | 0.981796984 | 1.001752101 | 5.882640187 | 5.872351236 |
| **SERPINE1** | 0.861482976 | 0.965907483 | 2.985897772 | 3.091287544 |
| **EVI2A** | 0.18612359 | 1.106358904 | 4.118406904 | 3.722487241 |
| **MMP17** | 0.329828901 | 1.197386469 | 2.170274514 | 1.812509637 |
| **TAGLN** | 0.089480133 | 0.852392161 | 6.258487547 | 7.342263146 |
| **MMP19** | 0.698515305 | 1.030424677 | 3.561231233 | 3.456081083 |
| **IKZF1** | 0.072600603 | 1.549180513 | 1.270151384 | 0.819885981 |
| **LRMP** | 0.75956537 | 1.050613625 | 2.65707614 | 2.529070703 |
| **RAC1** | 0.649333652 | 0.988466054 | 8.21617488 | 8.312045561 |
| **LAIR1** | 0.215066537 | 1.078118417 | 4.932642793 | 4.575232848 |
| **CCRL2** | 0.567285066 | 0.944442274 | 2.285704492 | 2.42016326 |
| **BCL2A1** | 0.310990187 | 1.126609255 | 3.397647364 | 3.015817017 |
| **CD97** | 0.260150469 | 1.122796018 | 4.210868541 | 3.750341536 |
| **LYN** | 0.577905554 | 1.061493663 | 4.448100975 | 4.19041689 |
| **TNFAIP3** | 0.560489546 | 1.071254007 | 3.425492777 | 3.197647575 |
| **CD37** | 0.478793462 | 1.049990714 | 5.157907638 | 4.91233643 |
| **IGF2R** | 0.59865818 | 1.06231845 | 4.333178726 | 4.078982838 |
| **BTK** | 0.536283671 | 1.059740666 | 3.232713791 | 3.050476304 |
| **TCF7L2** | 0.586608865 | 0.930614079 | 2.287846687 | 2.458426901 |
| **CD86** | 0.1419534 | 1.137076916 | 3.653576864 | 3.213130803 |
| **AIM2** | 0.070136288 | 1.459811822 | 3.582171454 | 2.453858367 |
| **MYO1F** | 0.434562565 | 1.058023854 | 4.436436371 | 4.193134543 |
| **NID1** | 0.266964807 | 0.85602789 | 2.822472725 | 3.297173793 |
| **MERTK** | 0.709840198 | 0.952378332 | 2.117433365 | 2.223311151 |
| **WIPF1** | 0.085882063 | 1.101919744 | 4.805135956 | 4.360695036 |
| **SELPLG** | 0.363401316 | 1.061677057 | 4.044380256 | 3.809426069 |
| **PLA2G7** | 0.271287308 | 1.132394505 | 3.157554714 | 2.788387527 |
| **COL1A2** | 0.051912982 | 0.81759782 | 7.382215867 | 9.029153073 |
| **CASP1** | 0.135814511 | 1.075300118 | 5.331383514 | 4.958042341 |
| **ADCY7** | 0.619398049 | 1.073407466 | 3.037482857 | 2.829757528 |
| **MMP2** | 0.092071612 | 0.810120381 | 4.894022613 | 6.041105402 |

**Supplementary Table 4. List of genes included in the gene-sets used for the anlayses**

| **Immunoscore** | **CYT score** | **GEP** | **CTL** | **Danaher et al** | **TLS related genes** |
| --- | --- | --- | --- | --- | --- |
| CD3D | PRF1 | CCL5 | GZMA | CD8A | IL1R1 |
| CD3E | GZMA | CXCL9 | GZMB | CD8B | CCL2 |
| CD3G |  | CD27 | CD8A |  | SDC1 |
| CD8A |  | CXCR6 | CD8B |  | CCL4 |
| CD8B |  | IDO1 | PRF1 |  | CCL3 |
|  |  | STAT1 |  |  | CD3E |
|  |  | TIGIT |  |  | IL16 |
|  |  | CD8A |  |  | ITGA4 |
|  |  | LAG3 |  |  | ITGAL |
|  |  | CD274 |  |  | CD86 |
|  |  | PDCD1G2 |  |  | CD4 |
|  |  | CD276 |  |  | IGSF6 |
|  |  | HLA-E |  |  | IL18 |
|  |  | NKG7 |  |  | CD40 |
|  |  | PSMB10 |  |  | STAT5A |
|  |  | HLA-DQA1 |  |  | CD38 |
|  |  | HLA-DRB1 |  |  | CCL5 |
|  |  | DMKLR1 |  |  | CCL18 |
|  |  |  |  |  | IL2RA |
|  |  |  |  |  | MS4A1 |
|  |  |  |  |  | CD68 |
|  |  |  |  |  | CCR2 |
|  |  |  |  |  | IL15 |
|  |  |  |  |  | CD200 |
|  |  |  |  |  | TRAF6 |
|  |  |  |  |  | CD80 |
|  |  |  |  |  | CXCL10 |
|  |  |  |  |  | CXCL9 |
|  |  |  |  |  | CCL20 |
|  |  |  |  |  | SGPP2 |
|  |  |  |  |  | IL1R2 |
|  |  |  |  |  | CD8A |
|  |  |  |  |  | PRF1 |
|  |  |  |  |  | ICAM3 |
|  |  |  |  |  | GNLY |
|  |  |  |  |  | SH2D1A |
|  |  |  |  |  | CD5 |
|  |  |  |  |  | CD28 |
|  |  |  |  |  | CTLA4 |
|  |  |  |  |  | TIGIT |
|  |  |  |  |  | ICOS |
|  |  |  |  |  | CCR5 |
|  |  |  |  |  | CD19 |
|  |  |  |  |  | CCR7 |
|  |  |  |  |  | IRF4 |
|  |  |  |  |  | CCL21 |
|  |  |  |  |  | CCL19 |
|  |  |  |  |  | CCR4 |
|  |  |  |  |  | LTA |
|  |  |  |  |  | TNF |
|  |  |  |  |  | TBX21 |
|  |  |  |  |  | CD40LG |
|  |  |  |  |  | CCL17 |
|  |  |  |  |  | CCL22 |
|  |  |  |  |  | FBLN7 |
|  |  |  |  |  | CXCR3 |
|  |  |  |  |  | IFNG |
|  |  |  |  |  | IL12B |
|  |  |  |  |  | ITGAD |
|  |  |  |  |  | PDCD1 |
|  |  |  |  |  | IL10 |
|  |  |  |  |  | CCL8 |
|  |  |  |  |  | CXCL11 |
|  |  |  |  |  | MADCAM1 |

**Supplementary Table 5. List of genes initially used for the differentially expressed gene test**

| **Macrophages/monocytes** | | | **lymphocyte infiltration** | **TGF-beta** | | **IFN-gamma** | | **wound healing** |
| --- | --- | --- | --- | --- | --- | --- | --- | --- |
| CORO1A | CD86 | NPL | ESR1 | MMP3 | IGFBP4 | IGSF6 | CRTAM | E2F1 |
| MNDA | HMHA1 | PLEK | CCL5 | MARCKSL1 | TNFRSF1A | LILRB2 | SLAMF8 | FOXM1 |
| CCRL2 | CTSL | CCL5 | CD19 | IGF2R | RAC1 | BTN3A3 | PSMB9 | PTTG1 |
| SLC7A7 | EVI2A | PTPRC | CD37 | LAMB1 | PXN | UBD | PTPN7 | CORO1C |
| HLA-DMA | TNFRSF1B | GNPTAB | CD3D | SPARC | PLAT | CXCL13 | SLAMF7 | FLNC |
| FYB | CXCR4 | SLC1A3 | CD3E | FN1 | COL8A1 | GNLY | BCL2A1 | LOXL2 |
| RNASE6 | LCP1 | HCK | CD3Z | ITGA4 | WNT8B | CXCR6 | TNFRSF17 | PLOD2 |
| TLR2 | SAMHD1 | NPC2 | CD79A | SMO | IGFBP3 | CTSC | CCL5 | PLAUR |
| CTSC | CPVL | C3AR1 | CD79B | MMP19 | RHOA | HCP5 | CCL8 | SDFR1 |
| LILRB4 | HLA-DRB1 | PIK3CG | CD8A | ITGB8 | EPHB4 | PIM2 | CCL13 | ESDN |
| PSCDBP | C13orf18 | DAPK1 | CD8B1 | ITGA5 | MMP1 | SP140 | CCL18 | MIF |
| CTSS | GIMAP4 | ALOX5AP | IGHG3 | NID1 | PAK1 | CCR7 | CCL19 | TAGLN |
| RASSF4 | SAMSN1 | CSF1R | IGJ | TIMP1 | MTA1 | CTSS | CXCL11 | TPM2 |
| MSN | PLCG2 | CUGBP2 | IGLC1 | SEMA3F | THBS2 | CYBB | SELL | MYL6 |
| CYBB | OSBPL3 | APOE | CD14 | RHOQ | CSPG2 | FCN1 | SAMSN1 |  |
| LAPTM5 | CD8A | APOC1 | LCK | CTNNB1 | MMP17 | TFEC | RTP4 |  |
| DOCK2 | RUNX3 | CD52 | LTB | MMP2 | CD59 | SEL1L3 | CLEC7A |  |
| FCGR1A | FCGR3A | LHFPL2 | MS4A1 | SERPINE1 | DVL3 | FYB | TAP1 |  |
| EVI2B | AMPD3 | C1orf54 |  | EPHB2 | RHOB | GBP1 | WARS |  |
| ADCY7 | MYO1F | IKZF1 |  | COL16A1 | COL6A3 | LAMP3 | PLA2G7 |  |
| CD48 | CECR1 | SH2B3 |  | EPHA2 | NOTCH2 | ADAMDEC1 | ZBED2 |  |
| ARHGAP15 | LYN | WIPF1 |  | TNC | BSG | GPR18 | NPL |  |
| ARRB2 | MPP1 |  |  | JUP | MMP11 | ICOS | RUNX3 |  |
| SYK | LRMP |  |  | ITGA3 | COL1A2 | GPR171 | VNN2 |  |
| BTK | FGL2 |  |  | TCF7L2 | ZYX | GZMH | CD3G |  |
| TNFAIP3 | NCKAP1L |  |  | COL3A1 | RND3 | GZMB | IL32 |  |
| FCGR2A | HCLS1 |  |  | CDH6 | THBS1 | GZMK | CD8B |  |
| VSIG4 | SELL |  |  | WNT2B | RHOG | BIRC3 | CD19 |  |
| FPRL2 | CASP1 |  |  | ADAM9 | ICAM1 | IFNG | CD86 | x |
| IL10RA | SELPLG |  |  | DSP | LAMA4 | IL2RG | AIM2 |  |
| IFI16 | CD33 |  |  | HSPG2 | DVL1 | IL15 | CD38 |  |
| ITGB2 | GPNMB |  |  | ARHGAP1 | PAK2 | IDO1 | CYTIP |  |
| IL7R | NCF2 |  |  | ITGB5 | ITGB2 | CXCL10 | LOC96610 |  |
| TBXAS1 | FNBP1 |  |  | IGFBP5 | COL6A1 | IRF1 | CD69 |  |
| FMNL1 | IL18 |  |  | ARHGDIA | FGD1 | ISG20 | CD79A |  |
| FLI1 | B2M |  |  | LRP1 |  | ITK |  |  |
| RASSF2 | SP140 |  |  | IGFBP2 |  | LAG3 |  |  |
| LYZ | FCER1G |  |  | CTNNA1 |  | LCK |  |  |
| CD163 | LCP2 |  |  | LRRC17 |  | LYN |  |  |
| CD97 | LY86 |  |  | MMP14 |  | CXCL9 |  |  |
| CCL2 | LAIR1 |  |  | NEO1 |  | NKG7 |  |  |
| FCGR2B | IFI30 |  |  | EFNA5 |  | TRAT1 |  |  |
| MERTK | TNFSF13B |  |  | ITGB3 |  | MGC29506 |  |  |
| CD84 | LST1 |  |  | EPHB3 |  | PLAC8 |  |  |
| CD53 | FGR |  |  | CD44 |  | POU2AF1 |  |  |
